# Bioinspired Rotation Microneedles for Accurate Transdermal Positioning and Ultraminimal-Invasive Biomarker Detection with Mechanical Robustness

**DOI:** 10.34133/2022/9869734

**Published:** 2022-03-07

**Authors:** Yilin Cai, Shiyi Huang, Zhinan Zhang, Jiazheng Zhang, Xingyue Zhu, Xiaoxiang Chen, Xianting Ding

**Affiliations:** ^1^State Key Laboratory of Mechanical System and Vibration, School of Mechanical Engineering, Shanghai Jiao Tong University, Shanghai 200240, China; ^2^State Key Laboratory of Oncogenes and Related Genes, Institute for Personalized Medicine, School of Biomedical Engineering, Shanghai Jiao Tong University, Shanghai 200030, China; ^3^Department of Rheumatology, Renji Hospital, Shanghai Jiao Tong University School of Medicine, Shanghai, China

## Abstract

Microneedle permits transdermal biosensing and drug delivery with minor pain. However, accurate microneedle transdermal positioning with minimal skin deformation remains a significant technical challenge due to inhomogeneous skin topology and discontinuous force applied to the microneedle. Here, we introduce bioinspired rotation microneedles for in vivo accurate microneedle positioning as inspired by honeybees' stingers. We demonstrate the benefits of rotation microneedles in alleviating skin resistance through finite element analysis, full-thickness porcine validations, and mathematical derivations of microneedle-skin interaction stress fields. The max penetration force was mitigated by up to 45.7% and the force attenuation rate increased to 2.73 times in the holding stage after penetration. A decrease in max skin deflection and a faster deformation recovery introduced by rotation microneedles implied a more precise penetration depth. Furthermore, we applied the rotation microneedles in psoriasis mice, a monogenic disorder animal model, for minimally invasive biological sample extraction and proinflammatory cytokine monitoring. An ultrasensitive detection method is realized by using only one microneedle to achieve cytokine mRNA level determination compared to commonly required biopsies or blood collection. Thus, rotation microneedles permit a simple, rapid, and ultraminimal-invasive method for subcutaneous trace biological sample acquisition and subsequent point-of-care diagnostics with minimal damage to both microneedles and skins.

## 1. Introduction

Microneedles have actively been studied for painless drug delivery [1–3] and biomarker diagnostics [4–6] through the transdermal route. Microneedles can perforate the skin effectively, especially the rigid barrier of the stratum corneum (SC), the outermost 10–20 *μ*m layer of the skin, without causing obvious pain [7, 8]. Therefore, microneedles are reported to enable accurate, reproducible results with better patient compliance and higher permeability [9, 10]. To effectively generate microchannels for clinical applications, microneedles should have an appropriate combination of mechanical strength and hardness to bear the skin resistance during insertion. Due to the extremely small size and limited material strength, microneedles are easily broken under the insertion force, leaving debris that causes potential injury to other organs [11, 12]. Studies have attempted to reduce insertion force by altering the microneedle structures such as changing the shape [13–15], sharpening the tip [16, 17], optimizing the diameter [11, 18], or using bioinspired structures [19–21]. Other reports also achieved penetration with low force by changing the motion mode, such as using a high-velocity insertion mechanism [22, 23] or combining vibration with insertion [24–26]. However, these approaches rely on a complicated manufacturing process with relatively high costs. Besides, they mainly address the mechanical behavior of microneedle while missing the skin responses, which leaves biosafety concerns unsettled for clinical translational application.

Meanwhile, the practical clinical applications of microneedles often require precise transdermal positioning, which would enable selective drug delivery or biomarker detection at particular skin layers [27]. However, current microneedles commonly suffer from inevitable high deviations in penetration depth, especially during the slow manual insertion process [28]. Conventional microneedle array produced on the flat substrate surface is of difficulty to insert on uneven skin surface completely, which leads to low penetration efficiency. In additional, due to the viscoelasticity and nonuniform topology of the skin, significant skin compression is required before the stratum corneum gets breached [5]. The compressed skin would perform a rapid rebound at rupture, causing a discontinuous subcutaneous position of the needle tip. As a result, insufficient skin penetration occurs and leads to practical difficulty in determining the actual penetration depth [29, 30]. Therefore, decreasing the skin deformation is essential to eliminate the error between net penetration and the needle displacement so that a short microneedle can effectively pierce the skin [21]. Some studies manipulated the skin's mechanical property variations by applying prestress [31], stretch, or vibration stimuli [28] to decrease the skin deflection. Although these strategies enhance penetration accuracy, they usually rely on cumbersome customized equipment with limited operation convenience.

Moreover, very few reports depict the microscopic mechanism of skin-microneedle interaction stress fields. The variance of force intensity and force direction during the manual insertion further contributes to the uncontrollability of skin deformation. Consequently, achieving precise and controllable penetration depth while respecting the mechanical strength constraint to ensure safety remains a significant technical challenge in clinical applications. In seeking of optimal microneedle design, we looked for inspiration from natural biomechanism of probe penetration. Insects are able to use their slender ovipositors or mouthparts to drill through harder substrates with special motion modes, implying a minimal net pushing force at the same time [32]. Especially, the drilling principle whereby the mouthpart structure performs rotating and torsions movements to open the skin is one of the basic principles of piercing mechanisms that can be distinguished [33]. The honeybees have stinger shaft which is found to rotate when inserting into tissues, and this helical penetration facilities a straight and easier insertion [34]. Such cyclic motion can reduce the deformation and strain on the surrounding substrate, which inspires the control of microneedles with cyclic rotation, with a likely reduction in damage along the needle insertion track. Similarly, piercing proboscis of Stomoxys is found to have prestomal teeth to rotate on the length axis of the proboscis during penetrating, which help them drill into or torn open the tissue [35]. Therefore, instead of altering the geometric structure which will influence the mechanical robustness and require higher manufacturing costs, we focus on the influence of motion profiles on insertion process.

**Figure 1:**
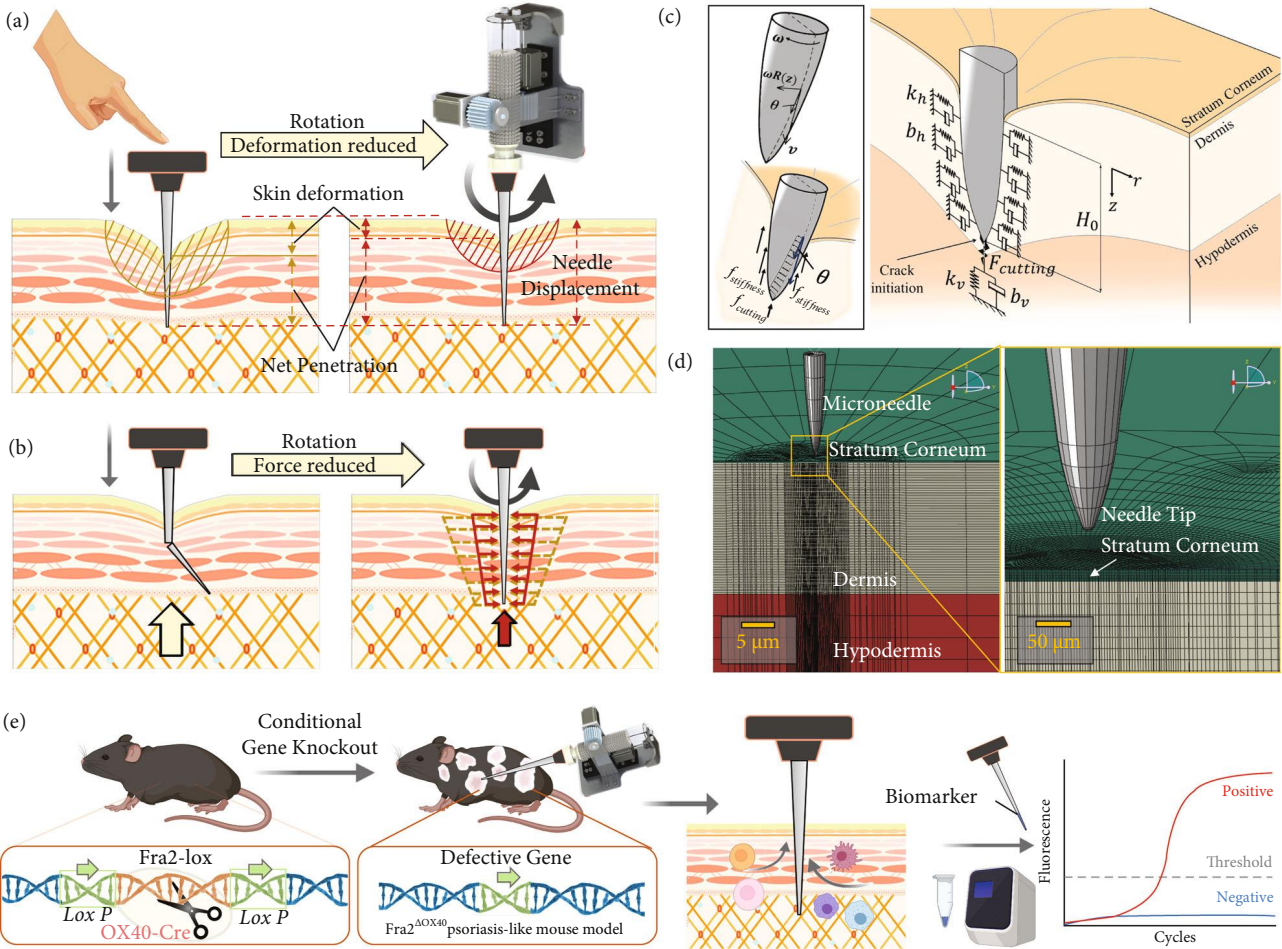
Schematic illustration of the rotation microneedle insertion process, mathematical theorem derivation, finite element simulation model, and ultrasensitive biosensing applications. (a) Direct manual perpendicular insertion would result in high skin deflection and low net penetration. Rotation microneedle insertion would decrease skin deflection and make net penetration closer to needle displacement. (b) Microneedles would be easily broken or bent due to the uncertainty of discontinuous force during the manual insertion. Rotation microneedles attenuate the side-wall force applied to needle (yellow arrows represent direct insertion and red arrows represent rotating insertion), which in turn decreases the axial friction force. (c) Theoretical model of microneedle total axial insertion force. Insertion force was divided into stiffness force, friction force, and cutting force. Microneedle-skin interaction stress field was built by the Kelvin-Voigt (KV) model. (d) Finite element model of the microneedle insertion simulation. (e) Rotation microneedle was used in subcutaneous biomarker detection of a psoriasis-like mouse constructed by Fra2-specific T-cell knockout using Cre-Lox recombination.

Herein, we developed bioinspired rotation microneedles to find an assistive method allowing precise and safe insertion and in turn improves the penetration efficiency (Figures 1(a) and 1(b)). A mathematical derivation theorem was constructed in this work to describe the microneedle-skin interface force portfolio (Figure 1(c)). A finite element model then numerically simulated the single microneedle insertion process and examined skin response (Figure 1(d)). Furthermore, a customized testing robotic system was developed to testify rotation microneedles with porcine skins. The skin resistance force was quantified for the force vs. time profile where the max force and force attenuation were paid specific attention to. The skin surface deformation was recorded synchronously as an intuitive reflection of skin resistance to external stimuli. Finally, we constructed a monogenetic disorder mice model for psoriasis and applied microneedle in proinflammatory cytokine detection of mice with psoriasis phenotype (Figure 1(e)). In this work, we systematically addressed the effect of the application of rotation on skin resistance during microneedle insertion in the level of mechanism, including insertion force, skin deformation, and penetration accuracy. We thus demonstrate a simple, rapid, and ultraminimal-invasive method for subcutaneous biomarker detection and analysis.

## 2. Results

### 2.1. Theoretical Stress Field Modeling Indicates Lower Insertion Force Using Rotation Microneedles

Herein, we define insertion force as the overall axial force applied to the microneedle tip due to skin resistance. The insertion force is divided into three components (Equation (1)): stiffness force *F*_*s*_, friction force *F*_*f*_, and cutting force *F*_*c*_ [36], as illustrated in Figure 1(d). Accordingly, the needle insertion process is divided into three phases: external contact deformation, surface puncture, and internal insertion movement [37]. The internal insertion is a cutting event that starts after a puncture where the new crack forms and propagates, on which we focus to analyze the influence of rotation.

(1) $$\begin{array}{c} {\text{F}}_{\text{total}}=F_{s}+F_{f}+F_{c}. \end{array}$$

The microneedle-skin interaction stress field was simulated using the Kelvin-Voigt (KV) model, which reflects the viscoelastic friction properties of the soft tissues [36, 38, 39]. A penetrator with a blunt tip would penetrate into the target by plugging while a sharp-tipped penetrator pushed the material in front of the tip away laterally during the penetration process. Hence, a conical tip microneedle is investigated. The KV model was applied both vertically and laterally to represent the behavior of tip plugging and sidewall pushing. As illustrated in Figure 1(d), taking the tip of the needle as the moving origin, the actual height of the needle insertion part is *H*_0_(*t*), and the direction of the needle axis is the *z-*axis (0 ≤ *z*≤*H*_0_). The clamping force of the unit area in the penetrated part *f*_*h*_(*z*) is

(2) $$\begin{array}{c} f_{h}\left(z\right)=b_{h}v_{ins}\tan\theta +k_{h}R\left(z\right), \end{array}$$

where *b*_*h*_ and *k*_*h*_ are horizontal stiffness and damping coefficients of the model, *v*_ins_ is the insertion velocity, *R*(*z*) is the needle radius at a depth of *z* under the skin surface with a base radius *R*_0_, and *θ* = arctan (*dR*(*z*)/*dz*). Since the horizontal component gets offset, the vertical component of friction force per unit area is written as

(3) $$\begin{array}{c} f_{fv}\left(z\right)=\mu f_{n}\left(z\right)\cos\alpha \cos\theta , \end{array}$$

where *f*_*n*_(*z*) = *f*_*h*_(*z*)cos*θ* is the unit area clamping force applied normally to the needle surface, and cos*α* indicates the combined direction of rotation velocity *ω* and insertion speed *v*. We have $\cos\alpha =v/\cos\theta /\sqrt{{\left(v/\cos\theta \right)}^{2}+{\left(R\left(z\right)\omega \right)}^{2}}$. Then, the total friction force is expressed as

(4) $$\begin{array}{c} F_{f}=\int_{0}^{R_{0}}f_{fv}\left(z\right)2\pi R\left(z\right)\text{d}R/\sin\theta =\int_{0}^{H_{0}}f_{fv}\left(z\right)2\pi R\left(z\right)\text{d}z/\cos\theta . \end{array}$$

When the microneedle is inserted into the skin without rotation, cos*α* degenerates to 1, and Equation (4) degenerates to

(5) $$\begin{array}{c} F_{f}=\int_{0}^{H_{0}}\mu \left[b_{h}v_{ins}\tan\theta +k_{h}R\left(z\right)\right]\cos\theta 2\pi R\left(z\right)dz. \end{array}$$

During the insertion process, the skin undergoes continuous deformation even if it is punctured. Thus, the vertical stiffness force is modeled by another pair of springs and dampers with the coefficient of *K*_*v*_ and *B*_*v*_. The stiffness force is

(6) $$\begin{array}{c} F_{s}=B_{v}v_{\text{ins}}+K_{v}H_{0}. \end{array}$$

Crack initiation is characterized by the fracture toughness *J*_*c*_ and crack-tip-opening displacement (CTOD). A crack initiates and grows when the energy released through crack extension equals or exceeds the fracture toughness [37]. Here, the cutting force in the vertical direction is a constant value expressed as

(7) $$\begin{array}{c} F_{c}=\frac{2\pi R\left(H_{0}\right)J_{c}}{\cos\theta_{0}}, \end{array}$$

where *θ*_0_ refers to the needle wall angle at the tip of the needle (*z* = *H*_0_). As a result, the theoretical model of total resistance force can be derived by Equation (1). Within the total insertion force, the friction force is the most critical attenuation term that indicates lower insertion force when rotation velocity gets higher.

However, when rotation motion is introduced, the microneedle undergoes a more complex force condition (Figure S1). To ensure that no break occurs during the insertion process, we clarified three failure modes theoretically with further mathematical derivation, namely, buckling failure, twisting failure, and maximum stress failure (Text ST1). Then, the theoretical relation between friction torque and positive pressure applied to the microneedle's tip was built. The critical axial force that the microneedle could withstand was introduced to give a unified description of the multicomponent failure mode. We considered the comprehensive stress distribution of the combined load of axial force and friction torque exerted to the microneedle (Text ST2). The load bearing capacity of microneedles was analyzed (Figure S2) under different geometric conditions, providing guidelines for microneedle design. It was found that the axial force was decreased effectively by rotation, and the max stress always occurred at the tip of the microneedle.

### 2.2. Finite Element Method Crossverifies Rotation Microneedle and Mitigates Skin Resistance Force and Deformation

**Figure 2:**
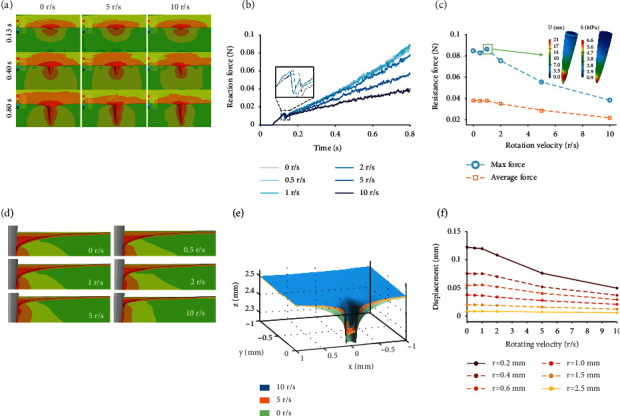
Finite element simulation results indicate that the microneedle resistance force and skin deformation are reduced by rotation. (a) Skin deformation and stress distribution (logarithmic) at *t* = 0.13 s, *t* = 0.4 s, and *t* = 0.8 s. These three time points represent the puncture point, half penetration, and complete penetration, respectively. The microneedle (hidden for better visualization) was inserted at an insertion velocity of 1.1 mm/s with rotation velocity of 0, 5, and 10 r/s (revolution per second). (b) Axial skin resistance force applied to the microneedles measured from the needle backend in the whole insertion process when rotating velocity is 0 r/s, 0.5 r/s, 1 r/s, 2 r/s 5 r/s, and 10 r/s. For better visualization, time definition starts from rotation rather than the contact moment. (c) Comparison of max and whole-process average insertion force at different rotating velocities. The effect of axial resistant force on the microneedle' stress (left) and displacement (right) is provided. (d) Cross-section skin deformation under microneedle rotating velocity of 0-10 r/s. (e) Deformation comparison of the entire central area where microneedle tip contacts with the skin. (f) Skin deformation along the radius distant 0.2 mm-2.5 mm away from the central insertion point at different microneedle rotation velocities.

The finite element method (FEM) was utilized to simulate the microneedle penetration into the skin to a fixed depth of 0.8 mm at the velocity of 1 mm/s. (Figure 1(e)). The von Mises (VM) stress distribution and deformation distribution show a trend of asymmetry and fluctuation when rotation velocity increased (Figure 2(a)). The portfolio of skin resistance force during microneedle insertion undergoes a process of continuous increasing, suddenly dropping, and then restored increasing (Figure 2(b)). The sudden drop indicated the puncture moment where the microneedle-skin contact area firstly reached an ultimate strength. The puncture force is robust to rotation velocity. At low rotation velocity of 0.5 and 1 r/s (revolution per second), the force curves coincide with no rotation (average force difference < 2%). Once the rotation velocity continued to increase, the max force was reduced by up to 54.9% ,and the average force was reduced by 43.0% (10 r/s to 0 r/s) (Figure 2(c)). Under such max force, the stress and displacement (subfigures in Figure 2(c)) were simulated using a static nonrigid model, indicating a max value of 6.6 MPa and 0.021 *μ*m concentration around the tip, respectively.

The FEM numerical simulation crossverifies the theoretical model that rotation induces lower insertion force. As for the skin deformation demonstrated in Figures 2(d) and 2(e), the increase in rotation velocity leads to a notable decrease of deformation after full penetration around the insertion point. Deformation gradient was also attenuated at higher rotation velocity along the radius (Figure 2(f)). The deformation at 0.2 mm from the center was reduced by 59.4% (10 r/s to 0 r/s), following the resistance force. At 10 r/s, difference of deformation from 0.2 mm to 2.5 mm was all smaller than 10 *μ*m, indicating that rotation readily reduces the area of skin being influenced by precompression. The FEM result provides the preliminary guidance of the influence of rotation on skin behavior for further experimental validation. Besides rotation, the influence of the geometries of microneedles was investigated (Figure S3), which also verifies the theoretical model.

### 2.3. Rotation Microneedles Reduce the Max Penetration Force and Enhance the Force Attenuation Ratio

**Figure 3:**
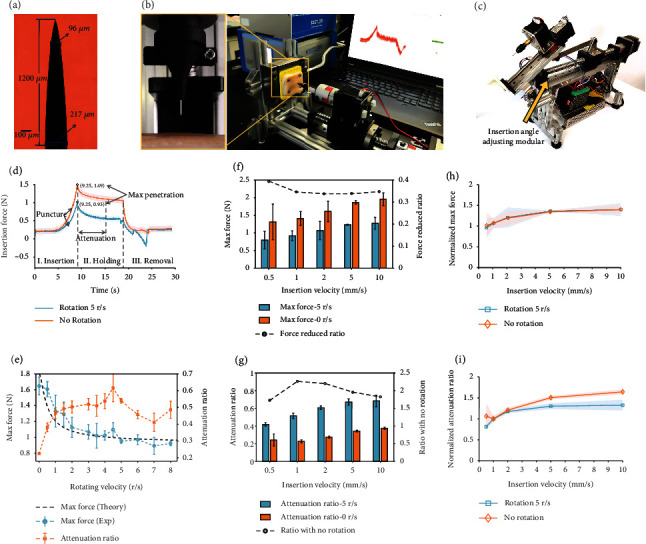
Rotation microneedles reduce max penetration force and enhance the force attenuation ratio. (a) Microscopic image of the tapered microneedle used in the experiment. (b) Home-developed robot-assisted microneedle insertion system with the insertion force measurement modular. (c) Robot-assisted microneedle insertion system with an insertion angle adjusting modular. (d) Axial skin resistance force applied to the tip of the microneedle during the insertion, holding, and removal process at rotation velocities of 0 r/s and 5 r/s and insertion velocity of 1 mm/s. Time definition starts from rotation rather than the contact moment with the skin. (e) Influence of rotation velocity on max penetration force (experimental and theoretical) and force attenuation ratio (experimental). The black dash line indicates the well-fitted theoretical maximum penetration force with the RMSE of 0.086 N. (f) Comparison of max penetration force F_pen_  at different insertion velocities. The black circle gives how much the F_pen_ was reduced compared to no rotation. (g) Comparison of attenuation ratio *η*_pen_^{1}^ under different insertion velocities. The black circle indicates how much the *η*_pen_^{1}^ was improved compared to no rotation. (h) Max penetration force normalized by the value at 1 mm/s-insertion velocity. (i) Attenuation ratio normalized by the value at 1 mm/s-insertion velocity. Colored background indicates 5% confidence interval. Data are presented as mean ± s.d. (*n* = 5).

Next, to estimate the insertion force profile when reaching a certain depth, the maximum penetration force was further investigated. Experimental validations were conducted by inserting microneedles into fresh porcine skin samples at different inserting and rotating velocities. Robot-assisted microneedle insertion system was developed to provide two degree-of-freedom (DOFs) motion of microneedles along with a force measurement modular (Figure 3) and an insertion angle adjusting modular (Figure 3(b)). The insertion force with respect to time was recorded in the three stages, namely, insertion, holding, and removal during microneedle penetration (Figure 3(d)).

We explored the effect of axial rotation (i.e., rotation velocity *n*_rot_), introduced to the microneedle insertion process, on skin resistance force. During the entire insertion, holding, and removal process, the skin resistance force at *n*_rot_ of 5 r/s was observed to be lower than that of direct insertion with no rotation (Figure 3(d)). At different *n*_rot_ ranging from 0.5 to 8 r/s, rotation induced a significant decrease in maximum penetration force *F*_pen_ (Figure 3(e)), by up to 45.7% (*P* < 0.01; 0.894 N under *n*_rot_ of 7 r/s in contrast to 1.647 N under no rotation). As the *n*_rot_ increased, *F*_pen_ experienced a relatively rapid decline process where max force decreased by 35.1% (*P* < 0.01) with *n*_rot_ increasing from 0 to 3 r/s. From 3 r/s to 8 r/s, however, the range of fluctuation in the percentage of reduction in force did not exceed 10%. Thus, we reasonably extrapolate to a lower bound that the penetration force will converge to if rotation velocity continuously increases. The bound is estimated by the average *F*_pen_ from 3.5 r/s to 8 r/s of 0.984 N, 40.3% reduction compared to no-rotation groups.

The force reduction trend and the lower bound were predicated by the mathematical model as a theoretical verification. Theoretical maximum penetration force was derived by Equation (1) where the stiffness and damping parameters were fitted by the experimental insertion process (the black dash line in Figure 3(e)). The root-mean-square error (RMSE) between theoretical and experimental results was 0.086 N, indicating a well-fitted result. To illustrate of lower bound, a critical saturation rotation velocity (SRV) is determined first by Equation (8).

(8) $$\begin{array}{c} \frac{F_{n_{c}}-F_{0}}{F_{\infty }-F_{0}}>0.95, \end{array}$$

where *F*_*n*_*c*__ is the penetration force at saturation rotation velocity, *F*_0_ is the force under no-rotation condition, and *F*_∞_ is the force when rotation velocity approaches infinite. For the porcine-based experimental model in this study, the SRV was calculated as 10.4 r/s, representing the smallest value to reach the lowest penetration force.

The force response in the holding stage was then clarified. After full penetration, the microneedle was stationary with only continuous axial rotation. It was worth noting that the skin resistance force constantly attenuates until gradually reaching a stable value in the 10 s-holding stage (Figure 3(d)). In clinical applications, the needle should stay under the skin for a certain amount of time, during which quicker force attenuation would reduce the possibility of needle damage. Thus, the force attenuation rate *η*_pen_^{*i*}^ was used to describe the long-acting influence of rotation on resistance force, especially after full penetration. *η*_pen_^{*i*}^ is defined as Equation (9).

(9) $$\begin{array}{c} \eta_{\text{pen}}^{\left\{i\right\}}=\frac{F_{\text{pen}}-F_{\text{holding}}^{\left\{i\right\}}}{F_{\text{pen}}}\times 100\%, \end{array}$$

where *i* denotes the attenuation time of interest during the entire holding stage, and *F*_holdling_^{*i*}^ denotes the skin resistance force at the attenuation time of interest *i* after the holding stage starts. Since the skin goes through a process of relaxation after compression, the *η*_pen_^{*i*}^ describes the ability of the skin to maintain the resistance forces when the microneedle gets fully penetrated. It tends to be hard to ensure enough holding time for the insertion force to stabilize for the various uncertainties in practical use. Here, we mainly focus on the force attenuated within one second. Dominant in the holding stage, the force maintenance ability illustrated by *η*_pen_^{1}^ could be easier to guarantee.

When *n*_rot_ increased from 0 to 3 r/s, the *η*_pen_^{1}^ showed a rapid corresponding increase from 22.54% to 51.95%. Then, the increasing trend slows down with max *η*_pen_^{1}^ reaching 61.58% (*n*_*rot*_ of 4.5 r/s). Overall, the attenuation ratio was positively correlated with the rotation velocity. Although the groups of 5 r/s, 6 r/s, and 7 r/s presented the opposite trend, they indicated a notable advantage in releasing skin resistance force. Like max penetration force, the attenuation ratio tended to increase to a relatively stable value. In conclusion, rotation was found to make skin resistance ability decline faster in microneedle insertion, leaving less stress to microneedles. Therefore, rotation endows microneedle higher mechanical robustness.

### 2.4. Force Reduction by Rotation Maintains at Different Insertion Velocities

Insertion velocity (*v*_ins_) is another crucial factor that influences the insertion force. It was reported that an increment in *v*_ins_ leads to a decrease in puncture force, but the benefit becomes less obvious when prestress was applied to the skin [37]. In our study, however, an increase in max penetration force *F*_pen_ was found as the *v*_ins_ increased from 0.5 mm/s to 10 mm/s under both rotation and no-rotation conditions. In this section, we would like to reveal a universal advantage of the rotation motion under different insertion velocity conditions.

As is illustrated in Figure 3(f), the *F*_pen_ at the *n*_rot_ of 5 r/s was all less than that under no-rotation conditions in the five groups of *v*_ins_ ranging from 0.5 mm/s to 10 mm/s. *F*_pen_ was reduced by a max value of 39.4% (at *v*_ins_ of  0.5 mm/s) and an average value of 35.3%. For attenuation ratio, an increase in *v*_ins_ would lead to a higher value no matter if there was rotation or not, which could be explained by a higher strain rate of the skin. However, the force attenuation ratio *η*_pen_^{1}^ was always improved at *n*_rot_ of 5 r/s compared with no rotation (Figure 3(g)). The *η*_pen_^{1}^ was improved by up to 2.26 times (at 1 mm/s) and by 1.99 times on average compared to the no-rotation condition. Both the max penetration force and attenuation ratio results have the consistent trend with those presented in Figure 3(e), reflecting the robustness to insertion velocities.

The above examination on insertion force included the combined effect of rotation and insertion. Next, we investigated if the effect of the 2-DOF-motion was also independent of each other. In Figures 3(i) and 3(j), max penetration force and attenuation ratio were normalized by the value under a *v*_ins_ of 1 mm/s. Comparison was made to show the consistency of variation trend with insertion velocity under rotation and no-rotation conditions. The variation trend when v_ins_ increased showed great consistency in increment between 5 r/s rotation and no-rotation conditions (Figure 3(h)). The average difference was 2.47%. Similarly, for the attenuation ratio, the average difference was 14.81% (Figure 3(i)). Consequently, the contribution of insertion velocity to penetration force increment would not be significantly affected by rotating motion.

### 2.5. Rotation Induces Attenuation in Skin Deformation

**Figure 4:**
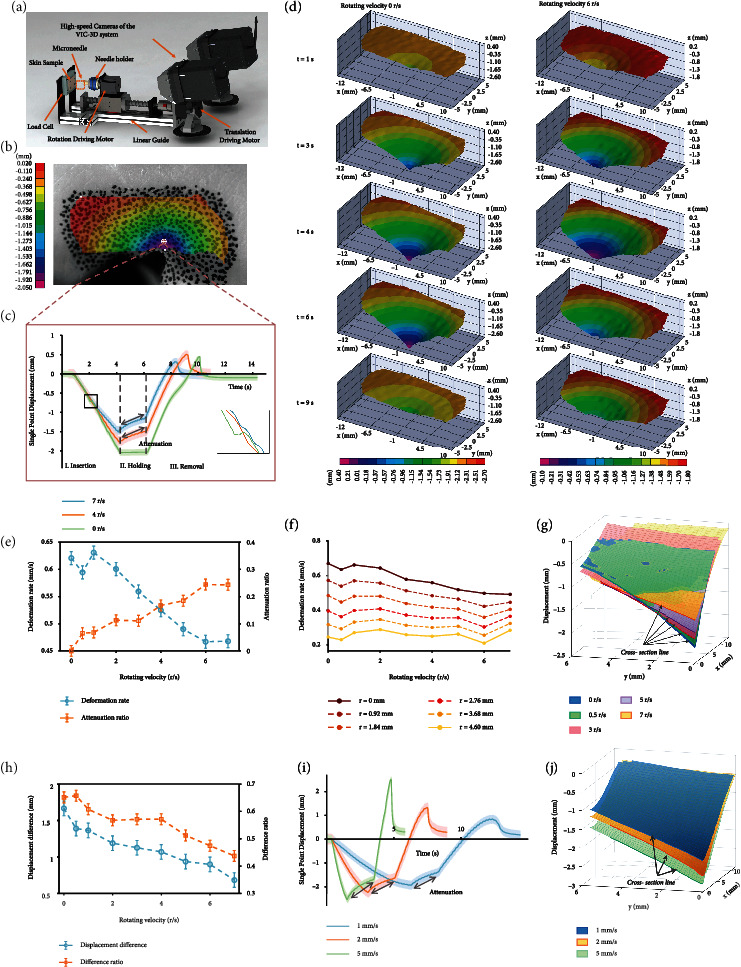
Skin surface deformation was attenuated by rotation microneedles. (a) Experimental setup of skin deformation reconstruction. High-speed stereo vision cameras and external light were adopted. Images were taken from an angle of 14 degrees against the normal direction of the skin. (b) Cloud map of the max deformation of the whole skin area at *n*_rot_ of 6 r/s and *v*_ins_ of 1 mm/s. The deformation was calculated as the absolute displacement perpendicular to the skin surface. Time definition starts from rotation (around 0.8 s before the contact moment with skin). (c) Deformation displacement of a single point on the skin with respect to time during insertion process at different rotation velocities of 0 r/s, 4 r/s, and 7 r/s. The single point is selected as the 20th pixel above the puncture point on the image. (d) Reconstruction of skin surface 3D deformation and comparison of the deformation at *t* = 1 s, 3 s, 4 s, 6 s, and 9 s after penetration begins at the rotation velocity of 0 r/s and 6 r/s. (e) Influence of *n*_rot_ on skin deformation rate ${\dot{z}}_{\text{pen}}$ and attenuation ratio *η*_disp_^{2}^. Data are presented as mean ± s.d. (*n* = 3). (f) Comparison of skin regional deformation at different rotation velocities along the radius from 0 to 4.6 cm. (g) Comparison of the max skin deformation at different n_rot_ after full penetration. The bold line at the edge of the surface represents the cross-section of the surface, which ends at the pixel point 4.6 cm away from the insertion point. One-quarter of the surface is displayed for clearer representation. (h) Influence of *n*_rot_ on skin displacement difference along the radius. The displacement difference was calculated as the displacement difference between the starting and ending pixel point of the cross-section line. (i) Insertion process and deformation comparison at *v*_ins_ of 1 mm/s, 2 mm/s, and 5 mm/s with *n*_rot_ of 5 r/s. Time definition starts from rotation (around 0.8 s before the contact moment with skin). The deformation rate at the deformation phase is 0.326 mm/s, 0.825 mm/s, and 2.178 mm/s, respectively. The attenuated deformation is 0.295 mm, 0.594 mm, and 1.151 mm, respectively. (j) Comparison of the max deformation at different insertion velocities of 1 mm/s, 2 mm/s, and 5 mm/s after full penetration.

The variation of both regional and single-point skin deformation under different rotation velocities was investigated in this section. The three-dimensional skin surface deformation was further reconstructed by the VIC-3D system (Figure 4(a)). The deformation was calculated as the absolute displacement perpendicular to the skin surface. During the fully penetration and needle removal process, the skin indicated noticeable in-plane compression and out-of-plane bulging (Figure S4). Due to the viewing angle limit, only the upper half of the deformed area was investigated. From the cloud map of maximum deformation moment, the skin deformation was uniformly distributed outward from the point of penetration (Figure 4(b) and S6). For a single pixel point on the skin, the deformation decreased when rotation velocity increased, and continuous rotation contributed significantly to the deformation recovery in the holding stage (Figure 4(c)). A drop at the puncture moment was also eliminated by superposing rotation motion (subfigure in Figure 4(c)), mitigating the discontinuity of insertion depth. Meanwhile, rotation motion was capable of reducing residual deformation of the skin, which was a permanent out-of-plane deformation of the skin after needle removal.

From the 3D reconstruction of the skin surface (Figure 4(d) and S5), the deformation gradient in the radial direction was almost identical, forming a series of circular contours centered on the insertion point expanding outward. Consistent with penetration force, the maximum displacement *z*_pen_ and attenuation ratio *η*_disp_^{*i*}^ during holding stage were used to represent the skin deflection behavior (Figure 4(e)). However, fluctuations in the actual penetration depth in different experiments could cause significant differences in deformation. Instead, the deformation rate ${\dot{z}}_{\text{pen}}$ (maximum displacement divided by the actual penetration time) was taken as a reflection of penetration accuracy.  *η*_disp_^{*i*}^ is defined as

(10) $$\begin{array}{c} \eta_{\text{disp}}^{\left\{i\right\}}=\frac{z_{\text{pen}}-z_{\text{holding}}^{\left\{i\right\}}}{z_{\text{pen}}}\times 100\%, \end{array}$$

where *z*_holding_^{*i*}^ denotes the skin deformation at the attenuation time *i* after the holding stage starts. Here, holding time was set as 2 s. As the rotation velocity increases, ${\dot{z}}_{\text{pen}}$ was found to be reduced by up to 24.7% (0.467 mm/s under *n*_*rot*_ of 7 r/s in contrast to 0.621 mm/s under no rotation). Also, the attenuation ratio *η*_disp_^{2}^ increased from 0 to 24.4% at 6 r/s. The influence of *n*_rot_ on ${\dot{z}}_{\text{pen}}$ and *η*_disp_^{2}^ reached a consistent agreement. Decrease of the skin deformation also means increasing the microneedle's net penetration depth given a specific insertion distance (closer to the targeted depth). The attenuation ratio was another reflection of skin resistance, indicating the positive effect of rotation on skin relaxation.

The deformation rate of the whole surface was then analyzed. The farther away the position was from the center, the less noticeable the influence of rotation could propose (Figure 4(f)). Taking the insertion point as the center, the increase of the *n*_rot_ could decrease the deformation of the whole skin surface within the 25 mm nradius (Figure 4(g)). Figure 4(h) shows the displacement difference along the radius, which implied the size of region being affected by the microneedle pore. The difference was 1.666 mm at 0 r/s and 0.685 mm at 7 r/s, showing a steady downward trend. Accordingly, the difference ratio with respect to the max displacement decreased from 65.14% to 43.73%. Although lower rotation velocities led to more considerable deformation, the area being affected tends to narrow. The narrowed affected region was reflected by ${\dot{z}}_{\text{pen}}$ of 0.232 mm/s at no rotation and 0.247 mm/s in average from 1 r/s to 7 r/s.

Similar comparisons were made when penetration was conducted at different *v*_ins_ with the same *n*_rot_ (Figure 4(i)), indicating increment of the *v*_ins_ enlarged the skin depression significantly (Figure 4(j) and S6). When *v*_ins_ increased from 1 mm/s to 5 mm/s, ${\dot{z}}_{\text{pen}}$ increased from 0.326 mm/s to 2.178 mm/s, and attenuation value increased from 0.295 mm to 1.151 mm (Figure 4(i)). Therefore, insertion velocity had a significant impact on skin deformation that higher *v*_ins_ would result in a larger deformation rate and deformation attenuation.

### 2.6. Rotation Microneedles Used for Proinflammatory Cytokine Detection of the Psoriasis-Like Mouse Monogenic Disorder Model

Subsequently, based on the findings of the skin response to microneedles, we applied rotation microneedles in detecting the biomarker expressions in the psoriasis-like mouse monogenic disorder model, including but not limited to essential proinflammatory cytokines. Psoriasis is a chronic inflammatory skin disorder affecting 1–3% of the world population [40]. The disease is generally believed to be associated with a complex network of immune cells and inflammatory cytokines such as tumor necrosis factor-*α* (TNF-*α*), interferon-*γ* (IFN-*γ*), interleukin-17A (IL-17A), and interleukin-23 (IL-23) [41]. However, current cytokine detection involves skin punch biopsies or vein blood collection, which could cause inevitable pain or invasiveness to the skin. The biomarker detection using microneedles could enable accurate sampling in specific skin layer while in an ultraminimal-invasive way. Moreover, the particularly thickened and hardened skin of the lesion caused by epidermal hyperplasia proposed increasingly requirements on the mechanical strength of microneedles. Thus, the advantage of rotating microneedles in alleviating skin resistance is prominent because it allows an easier penetration method for transdermal drug delivery or subcutaneous minimally invasive detection. Here, we set out to demonstrate a simple, rapid, and ultraminimal-invasive method for proinflammatory cytokine detection of psoriasis using rotation microneedle.

**Figure 5:**
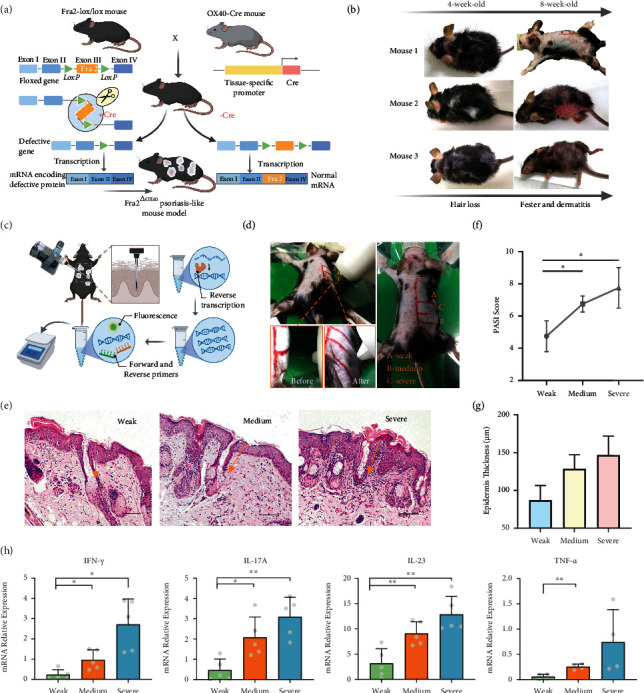
Single microneedle enables ultrasensitive proinflammatory cytokine detection of the psoriasis-like mouse. (a) Construction of psoriasis-like mouse model by Fra2 specific T-cell knockout using Cre-Lox recombination. (b) The lesions and depilation of the mouse are clearly observed from 4-week-old to 8-week-old, indicating the success of psoriasis-like model construction. (c) Workflow of microneedle-based biodetection involving in situ sampling, extraction, reverse transcription, and qPCR. (d) Microneedle penetration and sampling process of one representative gene knockout mice with psoriasis-like lesions. The lesional skin was divided into A (weak), B (medium), and C (strong) according to the severity of the inflammation. (e) H&E-stained section of mouse skin showing the epidermis thickness and the microchannel created by a single microneedle (indicated by yellow arrows). Scale bar, 100 *μ*m. (f) PASI score of the skin area. Data are mean ± s.d. (*n* = 5). PASI score was based on scoring erythema, scaling, and thickening with values ranging from 0 to 4 as follows: 0, none; 1, slight; 2, moderate; 3, marked; and 4, very marked. (g) Epidermis thickness of different lesional skin measured from H&E-stained section. Data are mean ± s.d. (*n* = 5). (h) The mRNA expression of IL-23, IL-17A, TNF-*α*, and INF-*γ* relative to the internal control gene. Data are mean ± s.d. (*n* = 5). ^∗^*P* < 0.05, ^∗∗^*P* < 0.01 by one-tailed unpaired *t*-test with Welch's correction.

Generally, proinflammatory cytokines were extracted from the lesions of psoriasis mice (Fra2 specific T-cell knockout mice) (Figures 5(a) and 5(b)) by microneedles, and mRNA levels of the cytokine were to be determined. The quantitative real-time PCR (qPCR) was then used to determine the expression level of the cytokine (Figure 5(c)). During the sampling process, the skin deformation, including compression, attenuation, and bulging, was observed in the mice model, similar to the ex vivo porcine skin experiment indicated (Figure 5(d)).

To evaluate severity of the inflammation of the back skin, hematoxylin and eosin (H&E) staining and the morphology of skin lesions were performed to acquire the clinical Psoriasis Area (Figure S7) and Severity Index (PASI) scoring (Figure 5(d)). The stratum spinosum extended downward, and the dorsal epidermis showed an obvious ridge-like shape, with an increased number of spinous layer cells. As demonstrated by PASI scores (Figure 5(f)) and the epidermis thickness (Figure 5(g)) and, the areas A, B, and C of the lesional skin were selected as the typical reflection of the medium, severe, and weak inflammation areas. Besides, H&E staining confirmed that the microneedles had penetrated the stratum corneum and perforated into the epidermal layer, which caused minor observable damage. Owing to the accurate force delivery of rotation microneedle, the penetration depth could be accurately controlled to around 160-180 *μ*m (Figure 5(e)). The micropuncture on the skin quickly became invisible as the skin recovered in 10 minutes (Figure S8). Such characteristic of rapid recovery reaffirms the minimally invasive detection.

Next, mRNA levels of proinflammation cytokines were detected. The qPCR results of IL-17A, IL-23, TNF-*α*, and IFN-*γ* were elucidated with microneedles in Figure 5(h). Compared with the weak inflammation area (area A), mRNA levels of all cytokines were robust in medium (area B) and severe (area C) lesional skin. The expression of IL-23 in the lesional skin with severe (area C) and medium inflammation (area B) was significantly upregulated (^∗∗^*P* < 0.01). In area C, the significant upregulation of IL-17A (^∗∗^*P* < 0.01) and IFN-*γ* (^∗^*P* < 0.05) can also be detected compared with area A. For the medium inflammation (area B), microneedles also detected the significantly upregulated mRNA expression of IL-17A (^∗^*P* < 0.05), IFN-*γ* (^∗^*P* < 0.05), and TNF-*α* (^∗∗^*P* < 0.01). Together, our results indicated the consistent pattern in the upregulation of the proinflammatory cytokine of psoriasis, which showed a simple and noninvasive tool in cytokine detection. In contrast to conventional blood drawing that involves painful sampling and time-consuming mRNA extraction, the microneedle method permits frequent and easy detection in disease diagnosis.

## 3. Discussion

Microneedles need to be accurately positioned into a targeted depth of skin with minimal damage to both microneedles and skin, which is essential to ensure the effectiveness and safety in clinical use. In this work, we proposed the bioinspired rotation microneedles and characterized skin response under the unidirectional continuous rotation stimuli by mathematical deduction, FEM numerical simulation, and experimental investigations. These three aspects of evidence together crossverify the rotation microneedles' superiority in improving penetration efficiency.

Although researchers have proved that geometric shape and material strength of microneedles played key roles in penetration efficiency, the dynamic needle-tissue interaction mechanism was often ignored. A special microneedle geometry tends reduce the insertion force or improve the adhesion by adding microstructure to its tip. However, these microstructures will in turn cause instability to its overall mechanical robustness, especially during the insertion process. In addition, the skin deformation depends more on the needle's motion profile during the dynamic interaction. The corresponding positioning accuracy is also a combined effect of geometry properties, external stimuli, and dynamic motion. Therefore, inspired from honeybees' stingers, we optimized the motion profile of microneedles towards lower insertion force and mitigated skin deformation. In this work, we balanced both microneedle and skin response at the same time. The microneedle force and skin deformation were investigated correspondingly during the whole cycle of inserting, holding, and retracting. Such rotation microneedles would be a more intuitive and feasible method without specialized design and manufacturing process. Moreover, rotation has the potential to facilitate the dominate characteristics of special microneedle's geometric structures during insertion process. For example, combined with microbarbs or protrusion arranged on microneedle tips, an altering of motion profile could further optimize the force response and controllability in needle insertion.

Max penetration force was paid particular attention to rather than the puncture point, which is the initial sudden drop in the measured force [11, 16]. In our study, there was a measurable drop in nonrotation force curve using a conical microneedle with tip diameter fewer than 5 *μ*m (Figure 3(a)). Once the rotation was added, the initial drop became less obvious (magnitude scale equivalent to noise) even at the low rotation speed. Such eliminated drop reduces the discontinuity of the insertion process which is another reflection of accuracy. However, a blunt microneedle can produce obvious puncture point and even reach it faster at relative low rotation velocities (Figure S3(c)). Before the puncture point, reaction force curves of different rotation velocities tend to coincide with each other, which means that apart from deformation, other factors contributed to the VM stress. Since the stiffness force *F*_*S*_ was induced only by deformation before puncture, the friction force applied on this area could be the key factor. Although some previous research abandoned the effects of friction along the needle shaft on needle-tissue interaction [42], we took it into careful consideration.

The lower bound of max penetration force could be mainly attributed to the friction force component in the total axial force. However, the reduction of penetration force is a combination of different factors. When considering the area of contact between the needle tip and the skin, the force to continue cutting the crack changes from the single axial pressure to a compound force that incorporates rotational friction. The drilling-like behavior also destroys the original unidirectional texture of the skin's fibrous tissue, thus reducing the axial friction. Energy could be another factor contributing to the reduced insertion force. The insertion process has been described as the interchange of energy between work performed by the needle, fracture of tissue, work against friction, and change in recoverable strain energy [43]. So, there is an increase in strain energy due to the compression of the skin. Rotation could also make the skin adhere to the needle surface and creates circumferential deformation, which brings changes in strain energy and kinetic energy.

Since vibration applied to microdevices was proved to be an effective method to reduce the insertion force, its function can be analogous to rotation. From axial vibration to rotation, the main difference lies in that vibration stimulation shift from vertical to lateral. Therefore, the skin shows a similar response. Firstly, mechanical impacts of the microneedle with the skin at high frequencies lead to an increase in the skin's dynamic stiffness. Secondly, lateral vibration pressure waves with the tissue fluid cause cavitation that destroys tissue [26]. Furthermore, acupuncture could also be analogous to the rotation microneedle that rotation strengthens the mechanical bond between needle and tissue, resulting in less slippage along the shaft of the needle during upward and downward needle movement [44].

In terms of microneedle behavior, the strength of the microneedle must be high enough to ensure that there is no fracture or buckling failure. The critical challenge here is determining whether the original large axial force brings more significant damage than the combined effect of reduced force and torque. We have developed the mathematical expression for the multicomponent failure mode and load bearing capacity of microneedles (Text ST1 and ST2). In FEM analysis of needle behavior under complicated force conditions (Figure 2(c)), a combination of reduced force and torque (Figure S3(c)) brings higher VM stress than using original force alone, but the stress was still less than the yield value of the material. Considering the geometric features, such thin and long needles always buckle first before reaching the yield stress. Consequently, reducing axial penetration force is vital in keeping microneedle safer, making it less possible to reach the buckling failure limit. Until now, we have given a detailed explanation of the superiority of rotation in microneedle mechanics.

In conclusion, by superposing rotation on the microneedle insertion process and exploring the skin response, we have achieved a precise penetration method with mechanical robustness. The subsequent experiments further verified and quantified the advantage of the rotation mode. The major findings of this study are summarized as follows:

1. Rotation significantly affects the skin resistance by reducing the penetration force and increasing the force attenuation rate in the holding stage after penetration. A lower bound of force and the corresponding saturation rotation velocity is identified. This effect will be maintained at different insertion velocities
2. The application of rotation leads to a decrease in max skin deflection and a faster recovery of skin deformation in the holding stage, which indicates a minor deviation between microneedle insertion displacement and actual penetration depth. In addition, the deformation difference along the radius could also be reduced, meaning a narrowed skin area is influenced by rotation microneedle insertion
3. Based on the gene knockout psoriasis-like mouse model, rotation microneedle demonstrates a simple, rapid, and minimal-invasive method for proinflammatory cytokine detection. Additionally, an ultraminimally invasive detection method is realized by using only one microneedle to achieve cytokine mRNA level determination compared to commonly required skin punch biopsies or vein blood collection

This study provides an in-depth understanding of skin biomechanics during a single rotation microneedle insertion, which is inspiring in novel microneedle devices development to enable efficient clinical applications at lower cost. Future research also proposes high expectations for integration multiple rotation microneedles into an array, considering the requirement of loading amount. Since single rotation microneedle-based cytokine detection proved its great potential in clinical application, promoting it to the form of array could bring much more benefits. For example, such array will accommodate the uneven force and geometric condition better. Therefore, our next plan includes (i) considering the influence of multiple rotation microneedles in skin-needle interaction mechanism, (ii) developing portable devices or medical robot systems that enable clinical biosensing or drug delivery based on multiple rotation microneedles, and (iii) changing rotating mode (e.g., bidirectional). We further expect these results to contribute to overcoming the mechanics' issues that microneedle currently faces and towards the next-generation clinical medical devices.

## 4. Materials and Methods

### 4.1. Parameters of the Finite Element Model

The human skin was found to be precisely modeled by a Neo-Hookean model, which was hyperelastic as well as incompressible [45]. An incompressible one-term Neo-Hookean model carried out in simulation writes as *U* = *C*_10_(*I*_1_ − 3), where the potential strain energy is represented by *U*. *I*_1_ is the first invariant of the stress tensor, and *C*_10_ is the parameter indicating the stiffness of the hyperelastic material. The finite element analysis was accomplished using Abaqus/Explicit solver (Abaqus 2016; Dassault Systemes Simulia Corp., Providence, RI, USA). A three-cylindrical-layer deformable skin model was built for finite element analysis, which consisted of a 20 *μ*m-thick SC, a 1.5 mm-thick dermis, and a 1 mm-thick hypodermis (Figure 1(f)). The stiffness coefficient of the SC and the dermis layer was finalized to be 10 MPa and 0.16 MPa, respectively. A linear elastic behavior was considered for hypodermis at the bottom layer with Young's modulus of 0.034 MPa and Poisson's ratio of 0.48. The radius of each cylindrical layer was set to 25 mm, much larger than the needle-skin contact area. This lateral boundary showed negligible displacement; so, the geometry truncation does not affect the numerical results.

The skin geometry was meshed with C3D8R element of growing size from the origin as the radius increases. VM stress's skin fracture criteria were controlled by deleting elements of which the VM equivalent stress reaches the ultimate strength [46]. The element deletion algorithm was developed in a subroutine. A 3D analytical rigid model was built for the penetrator. Boundary conditions were implemented at the base of the hypodermis [47]. Node-to-surface interface was enforced between the rigid body microneedle and the skin with a penalty contact method. The contact includes a friction coefficient of 0.42 and tangential behavior using the default Coulomb friction model. Mass scaling and enhanced hourglass controls were also activated to speed up simulation and prevent element crushing [23].

### 4.2. Preparation of Microneedles

For the convenience of installation and replacement, a commercial acupuncture needle (Beijing Daming Technology Co., Ltd, China) made of stainless steel (06Cr19Ni10) was used to substitute for a real microneedle. Such metal microneedle processes enough mechanical strength to accommodate different experimental conditions. The total length of the acupuncture length is 50 mm while the last half is the needle handle formed by helical coiling of the same needle material. A specific holder was designed for gripping the needle and also as a connector to the rotary actuation, leaving 15 mm (in length) of the microneedle for insertion. The needle tip curved conical geometry and formed a sharp end. From the sharp end, the diameter of the tip part of which the length is approximately 1.5 mm grows smoothly until reaching a constant value. The geometry of the microneedles from respective devices is visualized using optical microscopy (Figure 3(a)).

### 4.3. Ex Vivo Skin Sample

Considering the limited availability and the ethical difficulties associated with using ex vivo human skin, the porcine skin was used as an alternative to study microneedle insertion behavior due to its similar physiological and histological properties as compared to human skin [48]. Full-thickness foreleg skin samples were obtained from a freshly slaughtered pig (aged approximately 2–3 years), reared explicitly for food. To avoid altering the skin's biomechanical properties, full-thickness hairless skin was necessary [49]. About 5 mm thick subcutaneous adipose tissue was kept in skin samples as the support to be more accordant with the in vivo situation with the tissue support under the skin. As shown in Figure 3(b), we cut each skin sample into a cuboid (30 mm (width) × 70 mm (length) × 7 mm (height)), with four holes drilled with self-tapping screws located at four corners. Through these holes, bolts were used to fix the skin sample to the bottom holder, a connector to the load cell. The four bolts maintained a constant stretching state of the skin sample and would not influence the microneedle insertion behavior because their locations were far enough from the insertion point. After pre-treatment, the skin samples were stored at -20°C and were cleaned and returned to normal temperature before the insertion experiment.

### 4.4. Ex Vivo Microneedle Insertion Experiment

A custom experiment system was established to realize the insertion force measurement, needle micromoving drive, and rotation drive (Figure 3(b)). A load cell was placed vertically at the side end of the whole system, with a supporter and the skin sample fixed on it. The load cell is a multicomponent dynamometer (resolution: 0.002 N, Type 9256C2, Kistler, Switzerland) for measuring the three orthogonal components of a force and moment. The microneedle gripped by a holder was attached to a rotary actuation which was then mounted to the micromoving module (Figure 3(b)). The rotating and insertion velocity could be controlled within 0-10 r/s and 0.5-10 mm/s, respectively. The microneedle was inserted into the skin from the same initial position while the insertion distance keeps constant. After complete penetration, different holding times of 1 s, 2 s, 5 s, and 10 s were set to compare the mechanical responses under continuous rotation, followed by the retraction process at the same velocity. The sequence of testing at multiple locations was randomized. These locations were offset from one another by at least 2.5 mm to avoid the influence of microchannels that had already formed.

### 4.5. Ex Vivo Skin Deformation Measurement

Since the skin deformation is of a small area centered on the point of penetration, evaluating the deflections by measuring a point-to-point difference before and after penetration is far from enough. It was reported that the maximum radius of the skin deflection during needle penetration was less than 0.5 cm. Considering the small deflections that occurred in a tiny area and in a limited time, a noncontact measurement system VIC-3D 8 (Correlated Solutions, Inc., Irmo, SC, USA) was used to measure and visualize the full-field 3-dimensional displacement of the skin. Before experimentation, a random speckle pattern was painted manually in the measurement area as required by the system. The stereo camera was adjusted to keep the needle tip in the center of each camera's field of view. Then, a quick calibration procedure obtained the parameters necessary for software calculation. During microneedle indentation, the skin's surface was continuously imaged from an angle of approximately 14 degrees against the normal direction of the skin (Figure 4(a)).

### 4.6. Animal and Sample Extraction

A psoriasis-like mouse model was induced in an 8-week-old C57BL/6 male mouse with a knockout of Fra2. The Ethics Committee approved animal experiments for the Animal Experiments of Shanghai Jiao Tong University. All experimental manipulations were conducted in accordance with relevant guidelines and regulations.

The microneedle was mounted on custom equipment to control the rotation and insertion mode similar to that used in ex vivo experiment. In addition, an insertion angle adjustment modular is added in adaptation to the position of the mouse. During sampling, microneedle was inserted into the mouse skin at 3 mm/s with 0 or 2 r/s rotating velocities and maintained for 5 s. The mouse was anesthetized and fixed at four limbs. The insertion angle was set to be perpendicular to the mouse skin surface. The insertion distance is 2 mm. After microneedles being removed from the skin, 5 *μ*l TE buffer was immediately used to rinse the tip of microneedles for 1 min for sample extraction. The microneedles were sterilized with 95% ethanol and then irradiated with UV for 0.5 h. For each lesional area on each mouse, five microneedles were used to collect mRNA.

### 4.7. Histological Analysis

After the mice back skin tissues were fixed with 10% neutral formalin fix solution (E672001, BBI Life Science, China), the tissues were embedded in paraffin and then cut into 5 *μ*m sections. The skin tissues were stained using a hematoxylin and eosin staining kit with a standard procedure. Briefly, the slides were deparaffinated and stained in hematoxylin (H9627, Sigma-Aldrich, USA) for 10 min and dipped in ammonium hydroxide solution. Then, slides were stained in eosin (E4009, Sigma-Aldrich, USA), rehydrated using graded ethanol, and treated with phenazine methosulfate. The morphologies of skin lesions and microneedle-insertion wounds were quantified and assessed under an optical microscope (BX53M, Olympus, Japan).

### 4.8. Quantitative Real-Time PCR

Total mRNA was extracted from microneedle sampling from the back after euthanizing the mice. Then, cDNA was synthesized by reverse transcription using cDNA Synthesis Kit (11119ES60, Yeasen, China). The resulting cDNA samples were analyzed by quantitative real-time PCR using a SYBR Green assay kit (D7228, Beyotime, China) on LightCycler 96 (Roche, USA) according to the manufacturer's instructions. The PCR was performed in 20 *μ*l volumes, and the reaction master mix consisted of 10 *μ*L of 2× PCR Master Mix buffer, 0.5 *μ*L of each primer, 2 *μ*L of template DNA (used directly from microneedle extraction), and 7 *μ*L of sterile Milli-Q water. The cycling conditions comprised 40 cycles at 95°C for 10 s, 53°C for 10 s, and 72°C for 30 s using a single fluorescence measurement. The sequences of the real-time PCR primers were shown in Table S1. The gene GAPDH was used as an internal control. The rinsed solution of blank microneedle by TE buffer was set as negative controls.

### 4.9. Data Analysis

A custom MATLAB (MathWorks Inc., USA) program was used to analyze the biomechanical data including skin surface profile, actuator motion, and load cell data. In mouse experiment, statistics were performed in GraphPad Prism. The *P* values were calculated using one-way analysis of variance (ANOVA) with posthoc least significant difference multiple comparison tests to compare multiple groups or Student's *t*-test to compare two groups. The criterion of statistical significance was set at *P* < 0.05. The values were represented as mean ± standard deviation.

## Data Availability

All data used to support the findings of this study are available from the corresponding author upon request.

## Supplementary Materials

Supplementary Materials Text ST1: microneedle failure mode. Text ST2: comprehensive load bearing capacity of microneedles. Figure S1: theoretical model of the external load applied to rotating microneedle. Figure S2: influence of microneedle geometric conditions on their load bearing capacity. Figure S3: FEA results of microneedle insertion with different tip diameters and geometries. Figure S4: skin surface deformation at three moments during microneedle insertion: before puncture, full penetration, and needle removal. Figure S5: skin surface deformation process during microneedle insertion. Figure S6: single-point and whole surface skin deformation at rotating velocities of 0-7 r/s with inserting velocity of 1 mm/s and at insertion velocities of 1-5 mm/s with rotating velocity of 5 r/s. Figure S7: H&E-stained section of mouse lesional skin with different severity of the inflammation. Figure S8: optical images of that microwound on mouse skin recovery process. Table S1: primers used for real-time PCR analysis.
