# Supplementary material for: Bioinspired Rotation Microneedles for Accurate Transdermal Positioning and Ultraminimal-Invasive Biomarker Detection with Mechanical Robustness
Source: Research (Wash D C). 2022 Mar 7;2022:9869734. doi: 10.34133/2022/9869734 (PMC8924791; doi:10.34133/2022/9869734)
Supplement: Supplementary Materials — Text ST1: microneedle failure mode. Text ST2: comprehensive load bearing capacity of microneedles. Figure S1: theoretical model of the external load applied to rotating microneedle. Figure S2: influence of microneedle geometric conditions on their load bearing capacity. Figure S3: FEA results of microneedle insertion with different tip diameters and geometries. Figure S4: skin surface deformation at three moments during microneedle insertion: before puncture, full penetration, and needle removal. Figure S5: skin surface deformation process during microneedle insertion. Figure S6: single-point and whole surface skin deformation at rotating velocities of 0-7 r/s with inserting velocity of 1 mm/s and at insertion velocities of 1-5 mm/s with rotating velocity of 5 r/s. Figure S7: H&E-stained section of mouse lesional skin with different severity of the inflammation. Figure S8: optical images of that microwound on mouse skin recovery process. Table S1: primers used for real-time PCR analysis. [file 9869734.f1.zip › Cai_Supplementary_Material.pdf]

Research
A SCIENCE PARTNER JOURNAL
SPJ
SCIENCE PARTNER JOURNALS

Supplementary Materials for

Bioinspired Rotation Microneedles for Accurate Transdermal Positioning and Ultra-Minimal-Invasive Biomarker Detection with Mechanical Robustness

Yilin Cai[1]†, Shiyi Huang[2]‡, Zhinan Zhang[1]*, Jiazheng Zhang[1], Xingyue Zhu[1],
Xiaoxiang Chen[3] and Xianting Ding[2]*

1 State Key Laboratory of Mechanical System and Vibration, School of Mechanical Engineering, Shanghai Jiao Tong University, Shanghai, 200240, China.
2 State Key Laboratory of Oncogenes and Related Genes, Institute for Personalized Medicine, School of Biomedical Engineering, Shanghai Jiao Tong University, Shanghai, 200030, China.
3 Department of Rheumatology, Renji Hospital, Shanghai Jiao Tong University School of Medicine, Shanghai, China.

Correspondence should be addressed to Xianting Ding; dingxianting@sjtu.edu.cn and Zhinan Zhang; zhinanz@sjtu.edu.cn

†,‡: These authors contributed equally to this work.

Content of Supplementary Materials

Text ST1. Microneedle failure mode.

Text ST2. Comprehensive load bearing capacity of microneedles.

Figure S1. Theoretical model of the external load applied to rotating microneedle.

Figure S2. Influence of microneedle geometric conditions on their load bearing capacity.

Figure S3. FEA results of microneedle insertion with different tip diameters and geometries.

Figure S4. Skin surface deformation at three moments during microneedle insertion: before puncture, full penetration and needle removal.

Figure S5. Skin surface deformation process during microneedle insertion.

Figure S6. Single-point and whole surface skin deformation at rotating velocities of 0-7r/s with inserting velocity of 1 mm/s and at insertion velocities of 1-5 mm/s with rotating velocity of 5 r/s.

Figure S7. H&E-stained section of mouse lesional skin with different severity of the inflammation.

Figure S8. Optical images of that micro-wound on mouse skin recovery process.

Table S1. Primers used for real-time PCR analysis.

Supplementary Information Text

Text ST1. Microneedle failure mode

The failure of microneedles means the failure of the material due to excessive positive stress or shear stress. During the insertion process, it is necessary to ensure that the microneedles have high enough strength and stiffness to ensure that no fracture or buckling occurs during the whole process of puncture, delivery and extraction. Due to the small size of microneedles, they can be deformed or fractured at the slightest force, which can be harmful if it results in the entry of partially fractured microneedle fragments into the human body.

However, when the rotating motion is introduced into the microneedle insertion process, the force situation becomes more complex. Therefore, in this section, the failure mode of the microneedle is analyzed first. The cantilever beam model is used for physical modeling of the microneedle. As shown in Figure S1A, the force of the microneedle is assumed to be axial pressure P, transverse force F and friction torque T due to rotation. The shape of the microneedle is conical, and the diameter of the needle bottom is D_1, the diameter of needle tip was D_2. The research object here is limited to solid microneedles, so its diameter varies with the axial direction (x direction).

Buckling failure.

Because of the slender rod-like structure of microneedles, when they are subjected to excessive axial pressure, they may suddenly become bent and lose their original equilibrium in the linear form. Under such circumstance, the microneedles cannot continue to carry the load, i.e., instability occurs, generally before fracture failure. This section investigates the stability under specific loads through buckling analysis and determines the critical loads for structural destabilization.

Euler's formula is usually used to describe the critical load for elastic buckling and is valid only for "long rods". The ultimate compressive strength of the rod material is independent of the geometry, but this is valid only for "short" rods. For medium length rods, buckling occurs after the stresses in the rod exceed the proportional limit of the rod material and before the stresses reach the ultimate strength. In order to predict the force required to cause a buckling failure, the transition slenderness ratio (rod constant, C_c) defined by Equation (S1) is used to determine the failure mode[1].

$$C_c = \sqrt{\frac{2\pi^2 E}{\sigma_y}} \qquad (S1)$$

Where E is Young's modulus of material; σ_y is yield strength of material. The actual length-to-slenderness ratio of the rod is calculated from Equation (S2).

$$SR = \frac{L_e}{r_g} = \frac{KL}{r_g} \qquad (S2)$$

Where L_e is the equivalent length of a solid cylinder and $L_e = K \cdot L$; r_g is the equivalent radius of a solid cylindrical section and r_g=$D/4$; L is the actual length of microneedle; K is the end fixation coefficient of microneedles, which is 0.7 for fixed-pinned beams; D is the diameter of circular cross section. For the conical column with diameter varying with height, the equivalent diameter is

$$D_{equivalent} = D_{tip} + \frac{D_{base} - D_{tip}}{3} \qquad (S3)$$

Equation (S4) and Equation (S5) give analytical expressions for the critical load. The Euler's formula (Equation (S4)) should be used when the SR is greater than C_c, in which case the stylet is considered a 'long rod'. Whereas if the needles are short, the load predicted using Euler's formula is often greater than the actually buckling load. Then the Johnson formula should be used (Equation (S5)).

$$F_{cr} = \frac{\pi^2 EI}{{L_e}^2} \ (SR > C_c) \tag{S4}$$

$$F_{cr} = A\sigma_y \left[1 - \frac{\sigma_y \left(L_e/r_g\right)^2}{4\pi^2 E}\right] \ (SR < C_c) \tag{S5}$$

Where the end fixation factor K measures the degree of motion restriction at the end of the microneedle. K also determines when the transition from the 'long rod' analyzed by Euler's formula to the 'short rod' analyzed by Johnson's formula is made. According to Park[1] and Xenikakis[2], a K=0.7 makes the correspondence between theoretical and experimental data more accurate. In addition to comparing SR and C_c, the transition from Euler's region to Johnson's region can also be specified using an aspect ratio of 12:1. In most cases, the use of Johnson's formula best predicts the lapse rate because most microneedles used for drug delivery applications are less than 1000 μm and the use of Euler's formula will significantly over-predict the lapse rate.

Material yield failure

The lateral forces acting on the needles are considered below. The lateral forces may lead to structural failure when the maximum normal stress due to the shear momentum is greater than the yield stress. Since the microneedle is a cantilever beam, the normal stress is given by Equation (S6) using the Euler-Bernoulli beam theory.

$$\sigma_1 = \frac{F_{lateral} \cdot l \cdot c}{I} \tag{S6}$$

Where σ is the local stress perpendicular to the neutral axis at distance c. l the distance from the tip, i.e., where the lateral force F is applied. I is the moment of inertia of the needle (for circular cross-section, $I = \pi D^4/64$).

Since the diameter of the tapered microneedle varies axially, the maximum stress at the same cross-section should be at the point where the value of c is greatest at the radius of the cross-section. Therefore, the local stresses at different positions are a function of axial x, as shown in Equation (S7).

$$\sigma_1(\mathrm{x}) = \frac{32F_{lateral} \cdot (L - x)}{\pi D(x)^3} = \frac{32F_{lateral} \cdot (L - x)}{\pi \left(D_t + \frac{(D_b - D_t)}{L}(L - x)\right)^3} \tag{S7}$$

The function decreases with x, with $\sigma(0)$ being the largest, i.e., the largest when the needle root is present. Equation (S8) gives the maximum force that may cause lateral bending of a solid tapered microneedle when only transverse forces are considered.

$$F_{Bending} = \frac{\sigma_y \pi {D_b}^3}{32L} \tag{S8}$$

Where σ_y is the yield strength of the material, L is the length of the microneedle; D is the diameter. However, this description is inaccurate because positive stress is not only caused by bending, but also by pressure in the axial direction. Therefore, there is an expression for the second component of positive stress as follows.

$$\sigma_2(x) = \frac{F_{normal}}{A(x)} = \frac{4F_{normal}}{\pi\left(D_t + \frac{(D_b - D_t)}{L}(L-x)\right)^2} \quad (S9)$$

The total positive stress due to the transverse force and the forward pressure is

$$\sigma(\mathrm{x}) = \sigma_1(x) + \sigma_2(x)$$
$$= \frac{32F_{lateral} \cdot (L-x)}{\pi\left(D_t + \frac{(D_b - D_t)}{L}(L-x)\right)^3} + \frac{4F_{normal}}{\pi\left(D_t + \frac{(D_b - D_t)}{L}(L-x)\right)^2} \quad (S10)$$

Taking deviation

$$\sigma'(\mathrm{x}) = 0$$

$$x_{max} = L - \frac{16F_{lateral} - 4F_{normal}(D_b - D_t)D_t/L}{(32F_{lateral} + 4F_{normal}(D_b - D_t)/L)(D_b - D_t)/L}$$

Three cases of x_{max} should be considered next.

(a) If $x_{max} < 0$,

$$8F_{lateral}D_b + F_{normal}\frac{(D_b - D_t)}{L}D_b < 12F_{lateral}D_t$$

$\sigma(x)$decreases with x and the maximum occurs at the root of the microneedle.

$$[\sigma(\mathrm{x})]_{max} = \sigma(0) = \frac{32F_{lateral}L}{\pi {D_b}^3} + \frac{4F_{normal}}{\pi {D_b}^2} \quad (S11)$$

Therefore, in order to ensure that the microneedle will not be destroyed by the material yielding, it is necessary to ensure that $[\sigma(x)]_{max} \leq \sigma_y$, i.e.

$$\frac{32F_{lateral}L}{\pi {D_b}^3} + \frac{4F_{normal}}{\pi {D_b}^2} \leq \sigma_y$$

(b) If $x_{max} > L$,

$$4F_{lateral} < F_{normal}\frac{D_b - D_t}{L}$$

$\sigma(x)$ increases with x. The maximum value is at the tip of the microneedle and the transverse force effect is negligible.

$$[\sigma(x)]_{max} = \sigma(L) = \frac{4F_{normal}}{\pi {D_t}^2}$$

(c) If $0 < x_{max} < L$, then the maximum value of $\sigma(x)$ appears on the side wall of the middle section of the microneedle.

$$[\sigma(x)]_{max} = \sigma(x_{max}) = \frac{\left(\frac{(D_b - D_t)}{L} + 8\right)^2 \left((3F_{normal} - 3F_{lateral})\frac{(D_b - D_t)}{L} + 8F_{lateral}\right)}{108\pi D_t^2 \frac{D_b - D_t}{L}}$$

According to Equation (S1)-(S11), the distribution of the maximum stresses inside the microneedle can be analyzed for different transverse and axial forces (different penetration angles). When the penetration angle is small and the resulting transverse force is small, the maximum stress occurs at the tip of the microneedle, satisfying the case in (b) above.

Twist failure

We introduce a rotational motion during microneedle insertion, and the needle tip rotates against the skin surface during the actual process. The rotational torque due to the driving force or friction between the tip and the skin surface is considered. Figure S1B gives a schematic diagram of the tip of a needle under the action of the frictional torque looking down. The reverse pressure exerted by the skin is applied vertically at the tip of the needle, and the torque due to friction under this pressure can be obtained by integration. Here we assume a pressure distribution of $P(r)$.

The expression for the shear stress induced by this moment in the microneedle is given by Equation (S12).

$$\tau = \frac{TR}{I_P} \tag{S12}$$

Where τ is the shear stress; T is the rotational torque applied to the tip of the needle; R is the radius of the needle tip; I_P is the inertia moment of the cross section, $I_p = \pi D^4/32$.

Before the needle penetrates the tissue, we can assume that the external moment T is always constant. The expression for the critical load in different cases is given below, considering three geometric conditions and the force model.

(1) Flat-ended with uniform pressure

$$P = \frac{F_n}{A_{tip}} \tag{S13}$$

Where F_n is the axial force exerted on the microneedle tip; A_{tip} is the area of the tip of the microneedle. The Coulomb friction model is used here, where the friction force is established as being proportional to the positive pressure acting on the friction surface.

$$T = \int_0^{R_{tip}} \mu(P \cdot 2\pi \cdot r \cdot dr)r = \frac{F_n}{A_{tip}}\frac{2}{3}\mu R_{tip}^3 = \frac{1}{3}\mu F_n D_{tip} \tag{S14}$$

Where μ is the friction factor and D_{tip} is the diameter of the microneedle's tip. The variation of the shear stress with the axial length of the microneedle can be obtained by substituting it into Equation (S12).

$$\tau(x) = \frac{T}{W_P} = \frac{1}{3}\frac{\mu F_n D_{tip}}{W_P} = \frac{16}{3}\frac{\mu F_n D_{tip}}{\pi D(x)^3} \tag{S15}$$

Corresponding to the maximum shear stress that the microneedle material can withstand (yield shear stress τ_σ), the ultimate compressive stress $F_{n_{cr}}$ that causes shear failure of the microneedle can be given in Equation (S16).

$$F_{n_{cr}} = \left[\frac{3\pi\tau_\sigma D(x)^3}{16\mu D_{tip}}\right]_{min} = \frac{3\pi\tau_\sigma {D_{tip}}^2}{16\mu} \quad (S16)$$

The diameter $D(x)$, which varies with the length of the microneedle, is analyzed here. It is considered that the point where failure is most likely to occur is at the tip of the needle, which corresponds to the diameter D_{tip}.

At this point, a new critical axial force $F_{n_{cr}}$ is introduced, which corresponds to the condition of the rotating microneedle. In order to ensure that the microneedle does not fail under stress, it is necessary to consider the possible failures caused by the axial reaction force directly piercing the same skin. In addition to the critical buckling force and the critical pressure that causes the material to reach the yield strain, torsional shear failure through friction should be considered.

(2) Flat-ended with nonuniform pressure

In addition to the assumption that the microneedle's tip is subjected to a uniform pressure distribution, a more complex case is considered below to simulate a more realistic skin depression condition. It is argued that as the depth of penetration of the microneedle varies with the needle's diameter, there exists a circumferential distribution of skin depressions centered at the point of penetration and a specific distribution of the pressure acting on the microneedle.

Here the solution of the classical Boussinesq problem is used for analysis, which is the problem of determining the stress distribution of the normal pressure deformation of a nonlinear elastic half-space at the boundary of a rigid punch[1]. Boussinesq first considers the problem of determining the stress state in an isotropic elastic half-space under the action of a concentrated force perpendicular to a traction-free surface, and the solution of this problem can be found in several ways.

The first one is to simplify the problem to a boundary value problem in potential theory. When the half-space surface is subjected to normal forces only, the elasticity problem reduces to finding a single harmonic function with a single layer characteristic of the distribution over the plane region. Its intensity is proportional to the applied normal force. Finally, the solution of the concentrated force problem is recovered as a special case of the general normal load[3].

The second method is to simplify the problem to an edge-value problem in potential theory, when the half-space surface is subject only to a normal force. Beginning with the Kelvin solution of the point force inside the space, the distribution of dipole combinations (equivalent to the distribution of compression centers along the axis) is used to remove the linear action of the shear force perpendicular to the Kelvin force on the plane, thus recovering the Boussinesq solution[3].

The third approach involves applying integral transformation techniques to solve controlled partial differential equations that can be used to explicitly satisfy the direct applicability of the Boussinesq problem for traction boundary conditions[3].

With reference to Sneddon[3], since the axial stiffness force is caused by the viscoelastic properties of the skin during surface puncture, the contact model is used here to calculate the force-deformation response of the needle in contact with soft tissue. At the same time, the Hankel transform and the theory of logarithmic integral equations are applied to systematically solve the contact mechanics problem. The expression for the depth h of penetration of the microneedle into the skin is given by Equation (S17).

$$h = \int_0^1 \frac{f'(x)}{\sqrt{1-x^2}} dx \quad (S17)$$

The corresponding expression for the total combined needle reaction force, $F_{stiffness}$, is given by Equation (S18).

$$F_{stiffness}(z) = \frac{4GR}{1-\nu}\int_0^1 \chi(\mathrm{t})\mathrm{dt} \tag{S18}$$

Where the function $f(x)$ is specified as follows: with the needle tip as the origin, the needle tip curve has the expression $y = f(r)$, where $r = Rx$, so $f(0) = 0$; R is the radius of the contact circle; the physical meaning of h is the parameter of the depth of penetration of the needle tip (into the soft tissue); the meaning of z is as described in Figure 1C, indicating the axial coordinates of the penetration of the microneedle. The expression for the $\chi(t)$ function is

$$\chi(\mathrm{t}) = \frac{2}{\pi}\left[h - t\int_0^t \frac{f'(x)}{\sqrt{t^2 - x^2}}dx\right] \tag{S19}$$

Thus, the expression for stiffness force is

$$F_{stiffness}(z) = \frac{4GR}{1-\nu}\int_0^1 \frac{x^2 f'(x)}{\sqrt{1-x^2}}\mathrm{d}x \tag{S20}$$

However, in describing the shape of the needle, the above formula applies only to needle shapes that are continuous at the edge of the radius, whereas for flat-ended cylindrical punches, the needle appears discontinuous in derivative at the boundary R of the cylinder. The needle shape function is denoted by $f(x) = 0$. Since the flat-ended cylindrical tip is not smooth at the tip edge, h is the given amount, and the actual process is represented by the externally applied added microneedle feed[4].

$$\chi(t) = 2h/\pi \tag{S21}$$

The total reaction force is:

$$F_n = \frac{4GRh}{1-\nu} \tag{S22}$$

Where F_n is the axial stiffness force; $F_{stiffness}$ is the pressure acting on the microneedle; ν is the Poisson's ratio; G is the shear modulus; h is the depth of depression.

The stress distribution given by the Hankel transform and the theory of the logarithmic integral equation at this point is given by Equation (S23).

$$\sigma(r) = -\frac{2Gh}{\pi(1-\nu)}\frac{1}{\sqrt{R_{tip}^2 - r^2}} \tag{S23}$$

Where $\sigma(r)$ is the circumferential stress distribution at the skin-tip contact surface, positive in the direction of pressure on the skin below. R_{tip} is the radius of the tip of the needle (flat sided circle).

Set the constant $C_0 = \frac{2Gh}{\pi(1-\nu)}$, then the pressure distribution on the surface of the needle is shown in Equation (S24).

$$P(r) = C_0\frac{1}{\sqrt{R_{tip}^2 - r^2}} \tag{S24}$$

In order to compare the conditions of the axial critical force, the integrated overall reaction force is related to the pressure distribution as given in Equation (S25).

$$P(r) = \frac{F_n}{2\pi R_{tip}} \frac{1}{\sqrt{R_{tip}^2 - r^2}} \quad (S25)$$

When $r = 0, P(r) = F_n/2\pi R_{tip}^2$, which is half the size of the pressure assumed to be uniformly distributed. And as r increases, the value of $P(r)$ becomes larger. Similarly, the moment introduced by the friction of the needle tip is calculated as in Equation (S26).

$$T = \int_0^{R_{tip}} \mu(P \cdot 2\pi \cdot r \cdot dr)r = 2\pi\mu C_0 \int_0^{R_{tip}} \frac{r^2}{\sqrt{R_{tip}^2 - r^2}} dr$$

$$T = 2\pi\mu C_0 \frac{\pi R_{tip}^2}{4} = \frac{1}{8} F_n \pi \mu D_{tip} \quad (S26)$$

The variation of shear stress with the axial length of the microneedle is

$$\tau(x) = \frac{T}{W_P} = \frac{\pi}{8} \frac{\mu F_n D_{tip}}{W_P} = 2\frac{\mu F_n D_{tip}}{D(x)^3} \quad (S27)$$

Finally, calculate the limit pressure for uneven pressure distribution.

$$F'_{n_{cr}} = \left[\frac{\tau_\sigma D(x)^3}{2\mu D_{tip}}\right]_{min} = \frac{\tau_\sigma {D_{tip}}^2}{2\mu} \quad (S28)$$

A comparison of the uniform pressure distribution shows that the ultimate compressive stress leading to shear failure of the microneedles has the following ratio, with the critical force $F_{n_{cr}}$ being slightly higher for uniform pressure distribution.

$$\frac{F'_{n_{cr}}}{F_{n_{cr}}} = \frac{1}{2} / \frac{3\pi}{16} = 0.8488$$

(3) Conical needle tip with nonuniform distribution

In the model of a flat-topped circular planar needle tip, a special case is essential where the radius of the tip is so small that the contact between the tip and the skin can be approximated as a point contact. The indentation of the skin is closer to the sidewall of the conical microneedle, in which case a conical model is needed to analyze the distribution of the reaction forces given by the skin.

For a microneedle with a semi-cone angle of α, the shape of the microneedle is $f(x) = xR\ cot\alpha$. In fact, here R is the radius of the contact circle, there is no concept of the tip radius, and the depth of penetration of the cone is $h = \frac{1}{2}\pi R\ cot\alpha$.

The total counterforce is expressed as

$$F_{n-cone} = \frac{\pi G R^2}{1 - \nu} \cot\alpha = \frac{4G \tan\alpha}{\pi(1 - \nu)} h^2 \quad (S29)$$

And outside of the contact radius R, the shape of the skin surface is expressed as:

$$y(x) = \frac{2h}{\pi R}\left[R \sin^{-1}(x/R) - x + \sqrt{(x^2 - R^2)}\right] (x > R) \quad (S30)$$

The stress distribution within the contact radius R is

$$\sigma(x) = -\frac{2Gh}{(1 - \nu)\pi R} \cosh^{-1}(x/R)\ (0 \le x < R) \quad (S31)$$

$\cosh^{-1}(x/R) = 1/\cosh(x/R)$ where σ is actually σ_{zz}, and if downward orientation is positive, then the above equation represents the vertical upward stress. The transverse σ_{rz}, since the boundary condition is zero, is the component of the pressure applied to the surface of the microneedle in the direction perpendicular to the tapered surface.

The maximum radius considered here is the contact radius, denoted by R_{con}, and considering only the vertical distribution, there is a pressure distribution as given in Equation (S32).

$$P_n(r) = \frac{G}{(1-\nu)}\frac{\cot\alpha}{\cosh(r/R_{con})}\sin\alpha = \frac{G}{(1-\nu)}\frac{\cos\alpha}{\cosh(r/R_{con})} \tag{S32}$$

Similarly, it can be deduced that the torque due to friction is

$$\begin{aligned} T &= \int_0^{R_{con}} \mu\left(P_n(r)\cdot 2\pi\cdot r\cdot\frac{dr}{\sin\alpha}\right)r \\ &= 2\pi\mu\frac{G\cot\alpha}{(1-\nu)}\int_0^{R_{con}}\frac{r^2}{\cosh(r/R_{con})}dr \\ &= 2\pi\mu\frac{GR_{con}^3\cot\alpha}{(1-\nu)}\int_0^1\frac{x^2}{cosh\,x}dx \\ &\approx 0.512022\pi\mu\frac{GR_{con}^3\cot\alpha}{(1-\nu)} \end{aligned} \tag{S33}$$

We also have $F_{n-cone} = \frac{\pi GR^2}{1-\nu}\cot\alpha$ then variation of shear stress with microneedle axial length is

$$\tau(x) = \frac{T}{W_P} = 8.192352\mu\frac{F_{n-cone}R_{con}}{\pi D^3(x)} \tag{S34}$$

Finally, the limiting pressure under uneven pressure distribution is calculated to be：

$$F''_{n_{cr}} = \left[\frac{\tau_\sigma D(x)^3}{1.303853\mu R_{con}}\right]_{min} \tag{S35}$$

In most studies, the radius of contact between the conical punch and the soft tissue is analyzed in conjunction with finite element simulations because R_{con} has not yet been able to give a precise theoretical description. There is another approximation, where the contact radius is directly approximated by the microneedle radius at the deformed surface. In other words, it is assumed that the soft tissue is always in close proximity to all the needles that penetrate it.

If the wall angle is large and the tip top radius is also large, the main contact surface should be considered as the top. Thus, the planar model is applicable. However, when the wall angle or the tip top radius are also small, the microneedle contact surface is not only at the top and the conical model is applicable.

Text ST2. Comprehensive load bearing capacity of microneedles

In this section, we consider of comprehensive stress distribution of the combination load of axial force and friction torque. Considering that the penetration process is as straight as possible, the positive and shear stresses due to bending stresses are ignored.

The shear stress due to needle tip torque is:

$$\tau(x) = \frac{T}{W_P} = \frac{1}{3}\frac{\mu F_n D_{tip}}{W_P} = \frac{16}{3}\frac{\mu F_n D_{tip}}{\pi D(x)^3} \tag{S36}$$

It occurs at the edge of the cross section. At the same time, the positive stress due to axial pressure is expressed as Equation S37.

$$\sigma(x) = \frac{F_n}{A(x)} = \frac{4F_n}{\pi D(x)^2} \quad (S37)$$

Where $D(x) = \left(D_t + \frac{(D_b - D_t)}{L}(L - x)\right)$.

For each cross-section, the point of maximum stress occurs at the edge of the cross-section, so the principal stress at the edge of each face is given by Equation (S38).

$$\sigma_{max}, \sigma_{min} = \frac{\sigma(x)}{2} \pm \frac{1}{2}\sqrt{\sigma(x)^2 + 4\tau(x)^2} \quad (S38)$$

The two principal stresses are one positive and one negative. And the second principal stress is zero, so according to the fourth strength theory

$$\sqrt{\frac{1}{2}[(\sigma_1 - \sigma_2)^2 + (\sigma_2 - \sigma_3)^2 + (\sigma_3 - \sigma_1)^2]}$$

$$= \sqrt{\sigma^2 + 3\tau^2} = \sqrt{\left(\frac{4F_n}{\pi D(x)^2}\right)^2 + 3\left(\frac{16}{3}\frac{\mu F_n D_{tip}}{\pi D(x)^3}\right)^2} \quad (S39)$$

It needs to be guaranteed to be less than or equal to $[\sigma] = \sigma_y$

$$\left[\left(\frac{4F_n}{\pi D(x)^2}\right)^2 + 3\left(\frac{16}{3}\frac{\mu F_n D_{tip}}{\pi D(x)^3}\right)^2\right]_{max} \leq {\sigma_y}^2 \quad (S40)$$

$$F_n \leq \left[\sqrt{\sigma_y^2 \frac{3\pi^2}{16}\frac{D(x)^6}{3D(x)^2 + 16\mu^2 D_{tip}^2}}\right]_{min} \quad (S41)$$

The above force reaches the limit at the smallest radius, i.e. the tip of the needle.

$$(F_n)_{cr} = \sqrt{\frac{3}{3 + 16\mu^2}}\frac{\pi\sigma_y D_{tip}^2}{4} \quad (S42)$$

Therefore, the axial critical axial force under the combined action of the two forces is only related to the tip diameter. Damage is most likely to occur at the tip. We can analysis the influence of diameter on forces using material of stainless steel who has the properties of E=220MPa, σ_y=11.6MPa (Figure S2(a) and S2(b)). The material of Castable resin, a new type of material that can be used to make 3D printed microneedles (Figure S2(c)) is also analyzed. Castable resin has properties of $E = 200\text{GPa}$, $\sigma_y = 275\text{MPa}$. Since Johnson and Euler formulas apply to different geometry conditions, a transition point exists at a certain length of the microneedles. The material of stainless steel could not capture the transition point (Figure S2(a) and (b)) while Castable resin shows a clear intersection point (Figure S2(c)). Thus, the load bearing analysis performed on Castable resin provides a more comprehensive illustration of the theoretical model of the critical force.

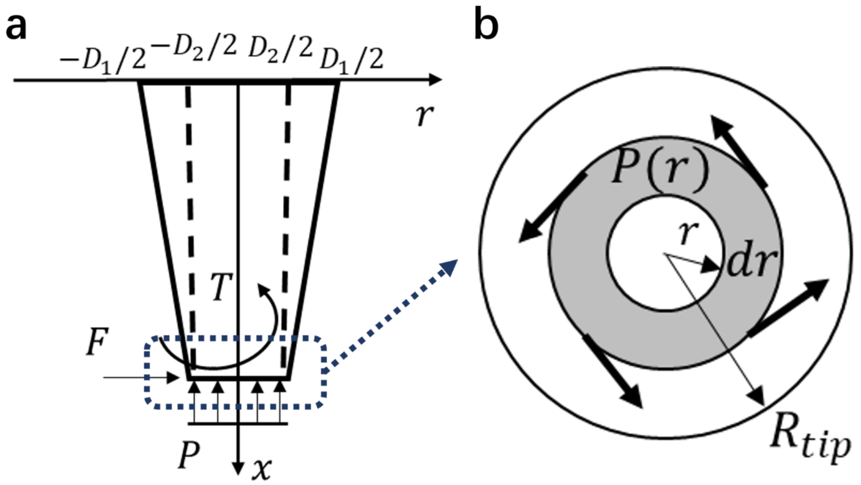

Figure S1. Theoretical model of the external load applied to rotation microneedle. (a) Cantilever beam model of the microneedles. (b) Frictional moment applied on the microneedle tip.

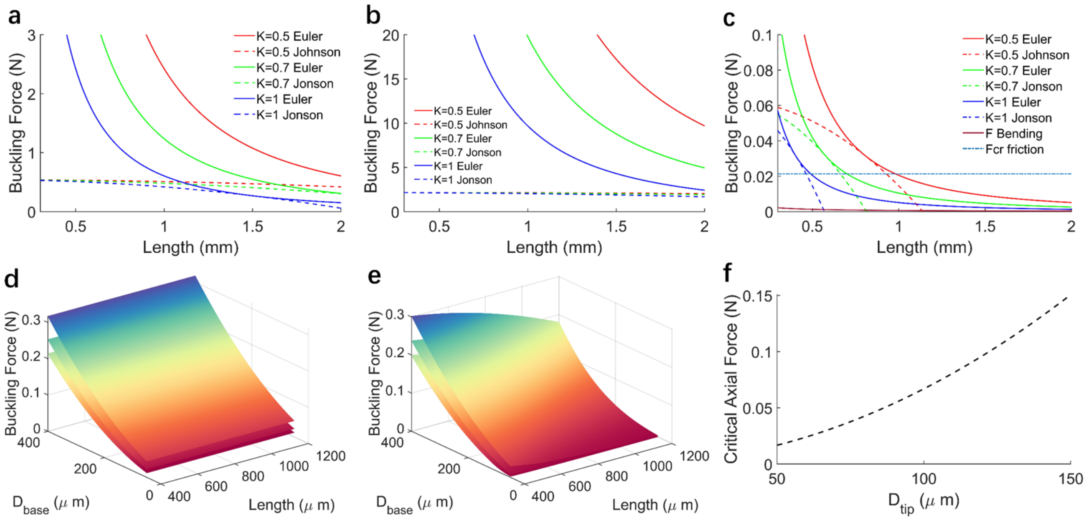

Figure S2. Influence of microneedle geometric conditions on their load bearing capacity. (a) The critical buckling force of microneedles made of stainless steel with D_{tip}=50 μm, D_{base}=50 μm. Johnson and Euler formulas were used for the entrie length changing range respectively. (b) The critical buckling force of microneedles made of stainless steel with D_{tip}=100 μm, D_{base}=100 μm. (c) Critical buckling force, lateral bending force and friction force of microneedles made of Castable resin with D_{tip}=50 μm, D_{base}=50 μm. The $F_{cr\ friction}$ was calculated using the flat-ended needle tip model under uniform pressure as Equation (S16). The $F_{bending}$ is the critical transverse force applied to microneedle tip, which is ignored due to the insertion direction that was normal to the skin surface. (d) The influence of length and based diameter on the final critical buckling force of the microneedle made of stainless steel. From down to up, the surface indicates different tip diameters with D_{tip}=30, 50, 80 μm. (e) The influence of length and based diameter on the final critical buckling force of the microneedle made of castable resin. From down to up, the surface indicates different tip diameters with D_{tip}=30, 50, 80 μm. (f) The influence of tip diameter on the critical force under the combined action of insertion and rotation given by Equation (S42).

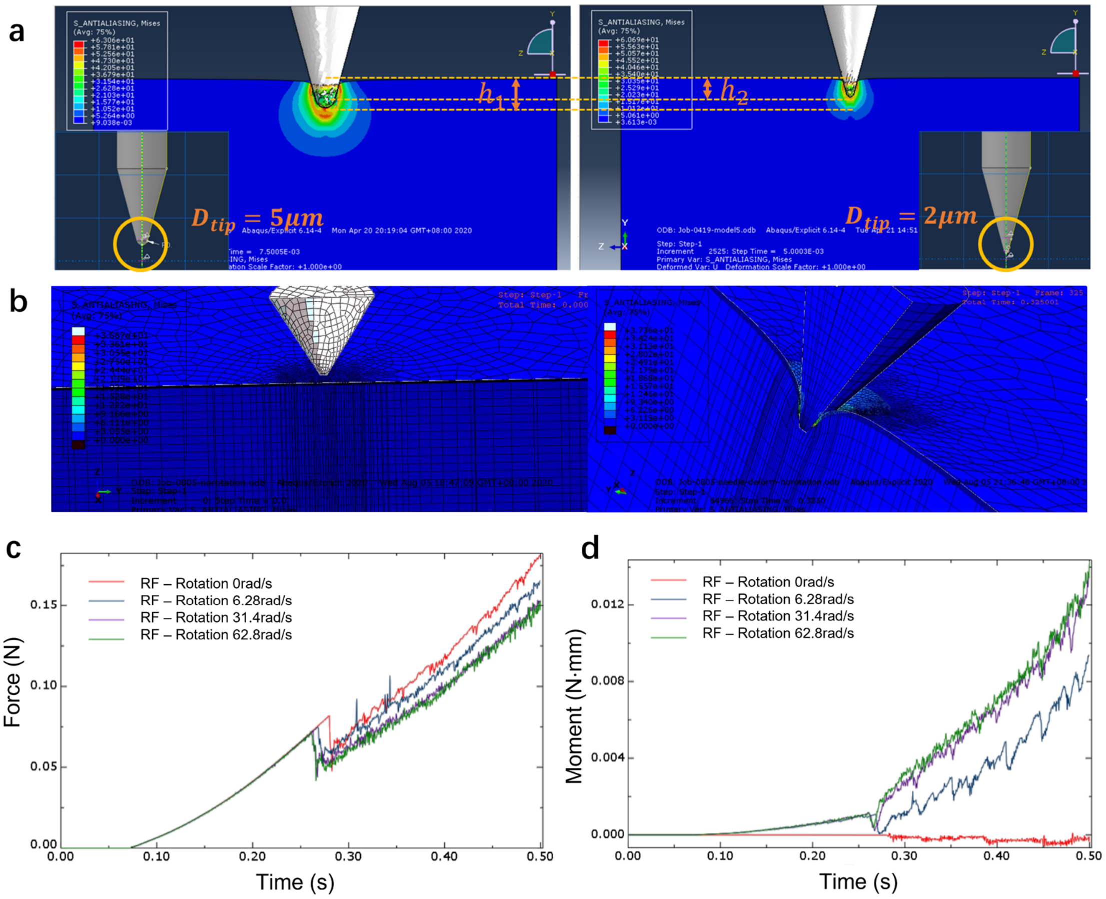

Figure S3. FEA results of microneedle insertion with different tip diameters and geometries. (a) Skin surface deformation at puncture point using microneedle with 5-μm and 2-μm diameter. The smaller diameter would result in skin smaller deformation. (b) Microneedle insertion simulation process with a blunt diameter with a 30 μm tip diameter. (c) Reaction force using blunt microneedles at different rotating velocities when the skin model was slightly compressible. The puncture force shows a significant decrease when rotating velocity increases. When rotating velocity increases from 5 r/s to 10 r/s (revolution per second), there is no obvious change in the insertion force which indicates a lower force limit. (d) Moment applied to microneedle by the skin friction due to the rotation. The moment also indicate a upper bound when rotation velocity increases.

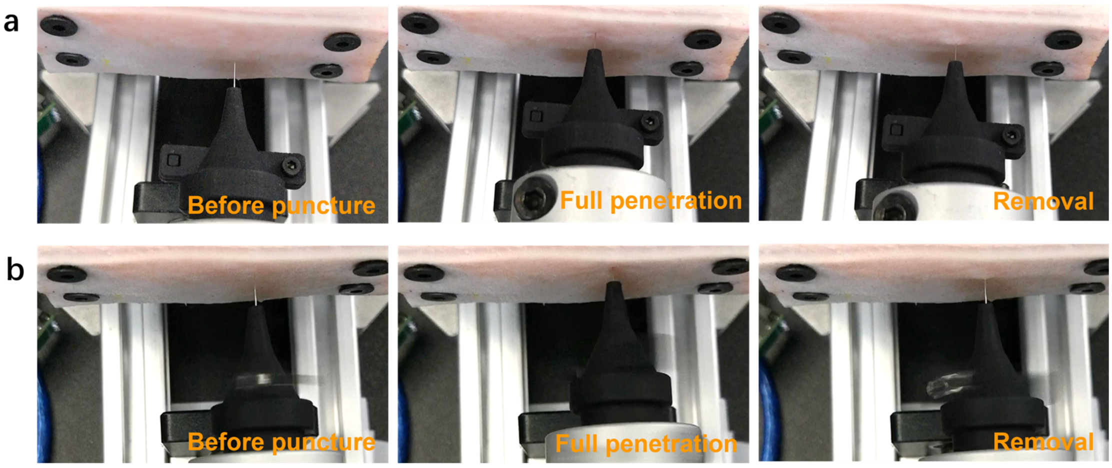

Figure S4. Skin surface deformation at three moments during microneedle insertion: before puncture, full penetration and needle removal. (a) Microneedle inserted at 1 mm/s and 0 r/s. (b) Microneedle inserted at 1 mm/s and 6r/s.

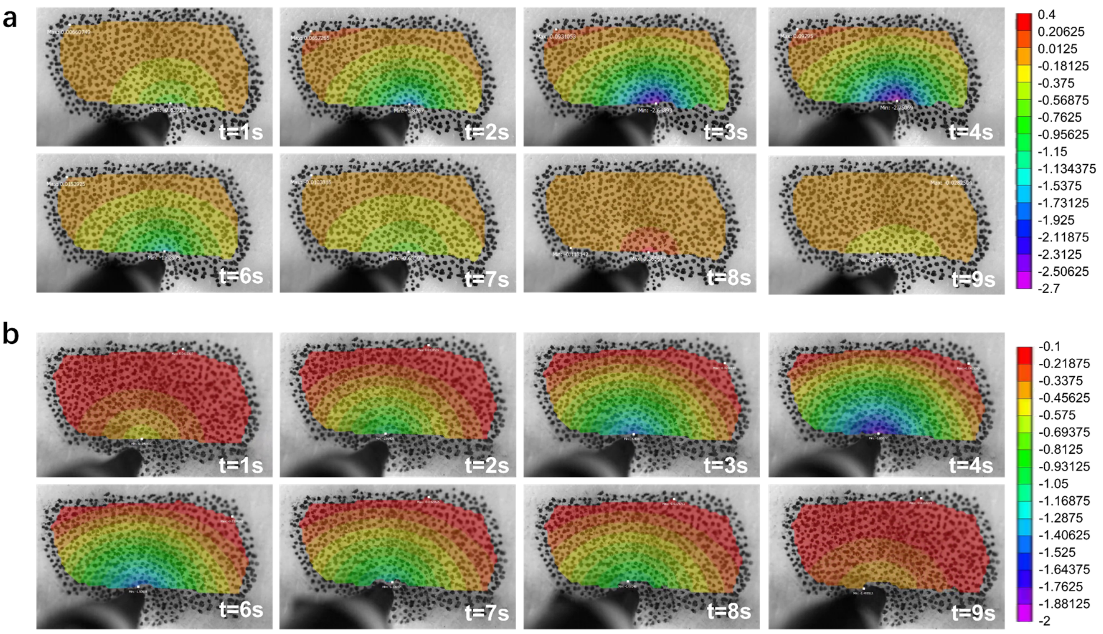

Figure S5. Skin surface deformation process during microneedle insertion. (a) Microneedle inserted at 1 mm/s and 0 r/s. (b) Microneedle inserted at 1 mm/s and 6r/s. The cloud map indicated the deformation displacement of the whole skin surface. Time definition starts from rotation (around 0.8s before the contact moment with skin).

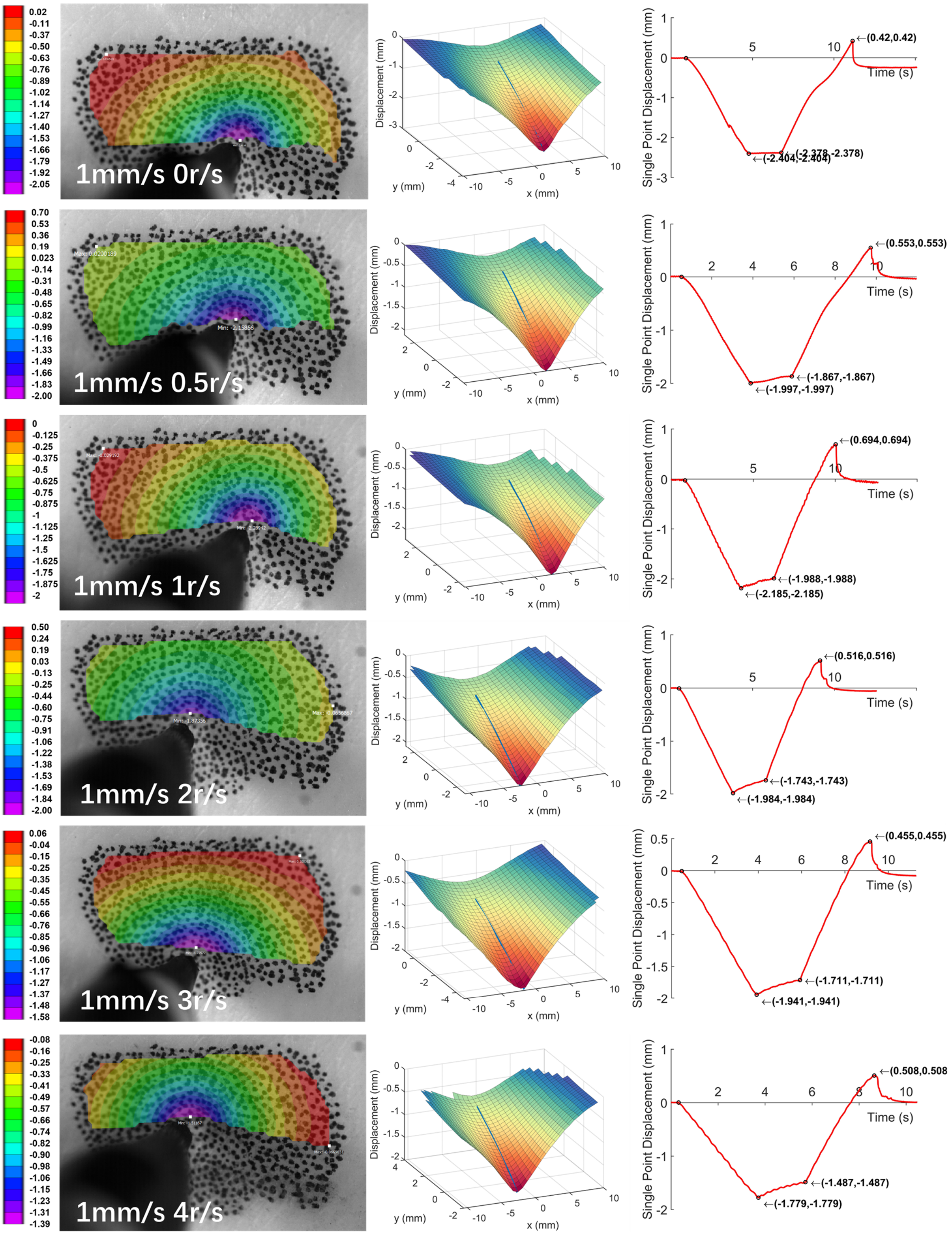
1mm/s 0r/s
1mm/s 0.5r/s
1mm/s 1r/s
1mm/s 2r/s
1mm/s 3r/s
1mm/s 4r/s
Displacement (mm)
y (mm)
x (mm)
Single Point Displacement (mm)
Time (s)
(0.42,0.42)
(-2.378,-2.378)
(-2.404,-2.404)
(0.553,0.553)
(-1.867,-1.867)
(-1.997,-1.997)
(0.694,0.694)
(-1.988,-1.988)
(-2.185,-2.185)
(0.516,0.516)
(-1.743,-1.743)
(-1.984,-1.984)
(0.455,0.455)
(-1.711,-1.711)
(-1.941,-1.941)
(0.508,0.508
(-1.487,-1.487)
(-1.779,-1.779)

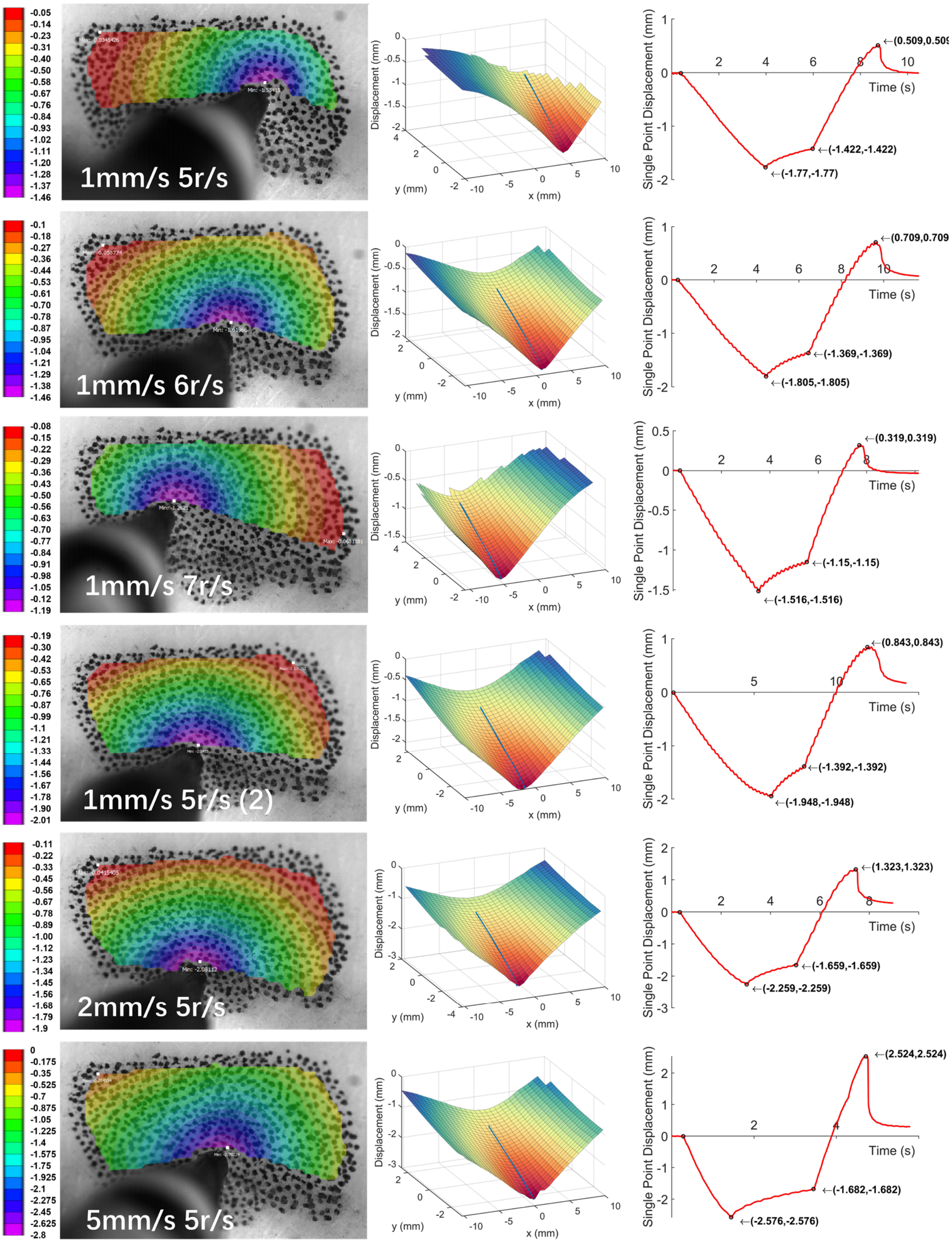

Figure S6. Single-point and whole surface skin deformation at rotating velocities of 0-7r/s with inserting velocity of 1 mm/s and at insertion velocities of 1-5 mm/s with rotating velocity of 5 r/s. From left to right: the photo of skin at max deformation moment; the reconstructed surface of max deformation; the displacement of single pixel point during the insertion process.

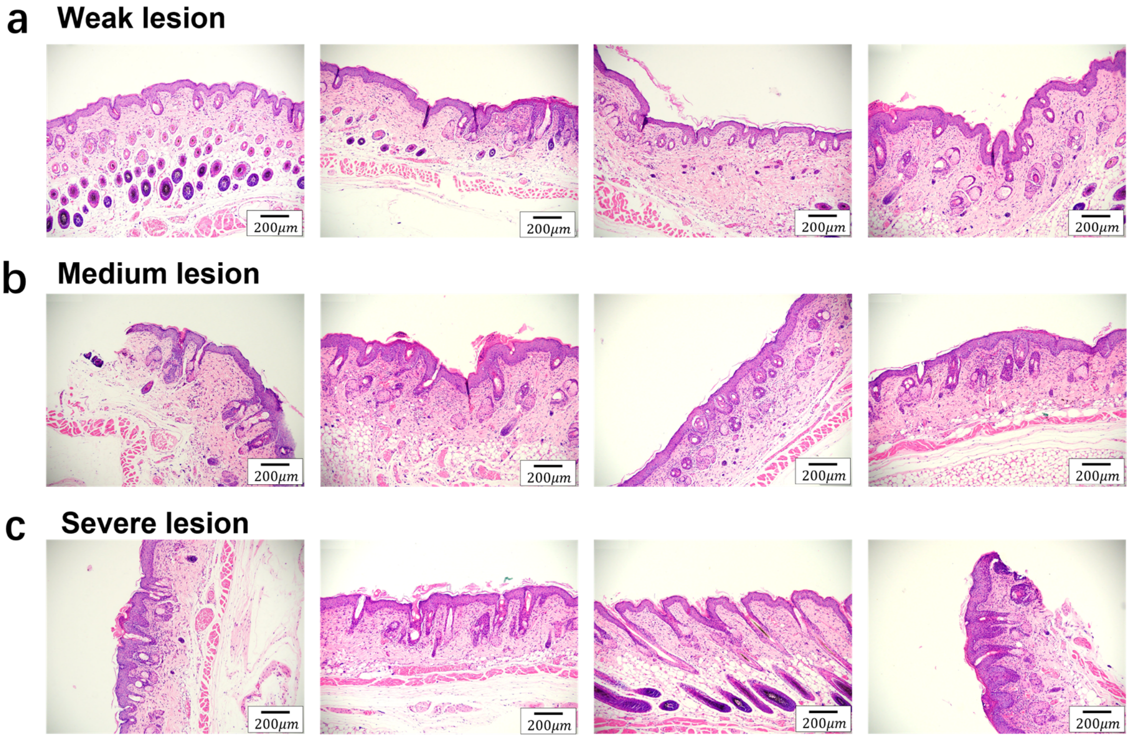

Figure S7. H&E-stained section of mouse lesional skin with different severity of the inflammation. (a) Weak lesion. (b) Medium lesion. (c) Severe lesion

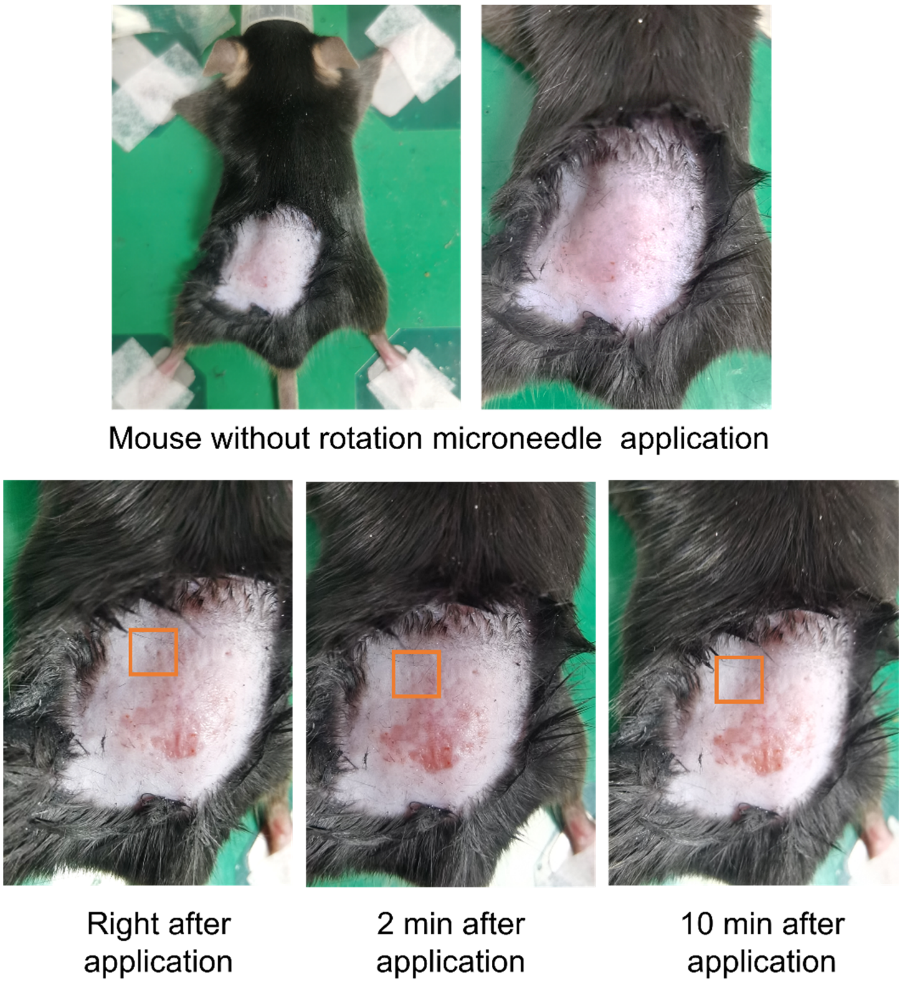

Figure S8. Optical images showing that micro-wound on mouse skin caused by the application of rotation microneedles quickly recovered in 10 minutes. (The application site is indicated by orange box.)

Table S1. Primers used for real-time PCR analysis

**Genes**	**Primer sequence**	
**IL-17A**	Forward	5’-CTCAGACTACCTCAACCGTTCC-3’
	Reverse	5’-ATGTGGTGGTCCAGCTTTCC-3’
**IL-23**	Forward	5’-CCCGTATCCAGTGT GAAGATG-3’
	Reverse	5’-CCCTTTGAAGATGTCAGAGTCA-3’
**IFN-γ**	Forward	5‘-GAACTGGCAAAAGGATGGTGA-3‘
	Reverse	5‘-TGTGGGTTGTTGACCTCAAAC-3‘
**TNF-α**	Forward	5’-GAGTGACAAGCCTGTAGCC-3’
	Reverse	5’-CTCCTGGTATGAGATAGCAAA-3’
**Filaggrin**	Forward	5’-GTTTCCAAACACATGGATCAAAT-3’
	Reverse	5’-TTTGAATCTTGTTGGTGTCT GTG-3’
**GAPDH**	Forward	5’-AGCTTGTCATCAACGGGAAG-3’
	Reverse	5’-TTTGATGTTAGTGGGGTCTCG-3’

References

[1] J.-H. Park and M.R. Prausnitz, “Analysis of Mechanical Failure of Polymer Microneedles by Axial Force.,” *The journal of the Korean Physical Society*. vol. 56, no. 4, pp. 1223–1227, 2010.

[2] I. Xenikakis, M. Tzimtzimis, K. Tsongas, et al., “Fabrication and finite element analysis of stereolithographic 3D printed microneedles for transdermal delivery of model dyes across human skin in vitro.,” *European Journal of Pharmaceutical Sciences*. vol. 137, no. June, p. 104976, 2019.

[3] I.N. Sneddon, “The relation between load and penetration in the axisymmetric boussinesq problem for a punch of arbitrary profile.,” *International Journal of Engineering Science*. vol. 3, no. 1, pp. 47–57, 1965.

[4] W. Liu, Z. Yang, P. Li, J. Zhang, and S. Jiang, “Mechanics of tissue rupture during needle insertion in transverse isotropic soft tissue.,” *Medical and Biological Engineering and Computing*. vol. 57, no. 6, pp. 1353–1366, 2019.
